# Local cascades induced global contagion: How heterogeneous thresholds, exogenous effects, and unconcerned behaviour govern online adoption spreading

**DOI:** 10.1038/srep27178

**Published:** 2016-06-07

**Authors:** Márton Karsai, Gerardo Iñiguez, Riivo Kikas, Kimmo Kaski, János Kertész

**Affiliations:** 1 Univ de Lyon, ENS de Lyon, INRIA, CNRS, UMR 5668, IXXI, 69364 Lyon, France; 2 Department of Computer Science, School of Science, Aalto University, 00076, Finland; 3 Centro de Investigación y Docencia Económicas, CONACYT, 01210 México D.F., Mexico; 4 Institute of Computer Science, University of Tartu, 50409 Tartu, Estonia; 5 Software Technology and Applications Competence Center (STACC), 51003 Tartu, Estonia; 6 Center for Network Science, Central European University, 1051 Budapest, Hungary; 7 Institute of Physics, Budapest University of Technology and Economics, 1111 Budapest, Hungary

## Abstract

Adoption of innovations, products or online services is commonly interpreted as a spreading process driven to large extent by social influence and conditioned by the needs and capacities of individuals. To model this process one usually introduces behavioural threshold mechanisms, which can give rise to the evolution of global cascades if the system satisfies a set of conditions. However, these models do not address temporal aspects of the emerging cascades, which in real systems may evolve through various pathways ranging from slow to rapid patterns. Here we fill this gap through the analysis and modelling of product adoption in the world’s largest voice over internet service, the social network of Skype. We provide empirical evidence about the heterogeneous distribution of fractional behavioural thresholds, which appears to be independent of the degree of adopting egos. We show that the structure of real-world adoption clusters is radically different from previous theoretical expectations, since vulnerable adoptions—induced by a single adopting neighbour—appear to be important only locally, while spontaneous adopters arriving at a constant rate and the involvement of unconcerned individuals govern the global emergence of social spreading.

Spreading of opinions, frauds, behavioural patterns, and product adoptions are all examples of social contagion phenomena where collective patterns emerge due to correlated decisions of a large number of individuals. Although these choices are personal, they are not independent but potentially driven by several processes such as social influence^1^, homophily^2^, and information arriving from external sources like news or mass media^3^. Social contagion evolves over networks of interconnected individuals, where links associated with social ties transfer influence between peers^4^. Several earlier studies aimed to identify the dominant mechanisms at play in social contagion processes^5^,^6^,^7^,^8^. One key element, termed behavioural threshold by Granovetter^6^, is defined as *“the number or proportion of others who must make one decision before a given actor does so”*. Following this idea various network models have been introduced^9^,^10^,^11^,^12^,^13^,^14^,^15^ to understand the threshold-driven spreading, commonly known as *complex contagion*^16^. Although these models are related to a larger set of collective dynamics, they are particularly different from *simple contagion* where the exposure of nodes is driven by independent contagion stimuli^17^,^18^. In addition, collective adoption patterns may appear as a consequence of homophilic structural correlations, where connected individuals adopt due to their similar interests and not due to direct social influence. Distinguishing between the effects of social influence and homophily at the individual level remains as a challenge^19^,^20^. Furthermore, in real social spreading phenomena all these mechanisms are arguably present. However, while in the case of homophily the adoption behaviour is only seemingly correlated, and for simple contagion only the number of exposures matters, in complex contagion the fraction of adopting neighbours relative to the total number of partners determines whether a node adopts or not, capturing the natural mechanisms involved in individuals’ decision makings^21^,^22^,^23^. Due to this additional complexity, threshold models are able to emulate system-wide adoption patterns known as global cascades. Regarding the effects of social influence several assumptions have been proposed about its functional dependency on the number of influencers. While Granovetter and others^6^,^9^ suggest a simple linear correspondence, e.g. observed in large techno-social systems^21^, from the theory of Latané^24^ one could derive a non-linear dependency as recently demonstrated by small-scale online experiments^1^.

Behavioural cascades are rare but potentially stupendous social spreading phenomena, where collective patterns of exposure emerge as a consequence of small initial perturbations. Some examples are the rapid emergence of political and grass-root movements^25^,^26^,^27^, fast spreading of information^12^,^28^,^29^,^30^,^31^,^32^,^33^,^34^ or behavioural patterns^35^, etc. The characterisation^34^,^36^,^37^,^38^,^39^,^40^ and modelling^9^,^41^,^42^,^43^ of such processes have received plenty of attention and provide some basic understanding of the conditions and structure of empirical and synthetic cascades on various types of networks^44^,^45^,^46^,^47^. However, these studies commonly fail in addressing the temporal dynamics of the emerging cascades, which may vary considerably between different cases of social contagion. Moreover, they have not answered why real-world cascades can evolve through various dynamic pathways ranging from slow to rapid patterning, especially in systems where the threshold mechanisms play a role and social phenomena spread globally. Besides the case of rapid cascading mentioned above, an example of the other extreme is the propagation of products in social networks^18^, where adoption evolves gradually even if it is driven by threshold mechanisms and may cover a large fraction of the total population^21^. This behaviour characterises the adoption of online services such as Facebook, Twitter, LinkedIn and Skype (Fig. 1a), since their yearly maximum relative growth of cumulative adoption^48^ (for definition see Appendix) is lower than in the case of rapid cascades as suggested e.g. by the Watts threshold (WT) model.

To fill this gap in the modelling of social diffusion, here we will analyse and model real-world examples of social contagion phenomena. Our aim is to identify the crucial mechanisms necessary to consider in models of complex contagion to match them better with reality, and define a model that incorporates these mechanisms and captures the possible dynamics leading to the emergence of real-world global cascades. We follow the adoption dynamics of the Skype paid service “buy credit” for 89 months since 2004, which evolves over the social network of one of the largest voice over internet providers in the world. Data includes the time of first payment of each user, an individual and conscious action that tracks adoption behaviour. In addition we follow the “subscription” service over 42 months since 2008 (for results see Supplementary Information [SI]). In contrast to other empirical studies where incomplete knowledge about the underlying social network leads to unavoidable bias^21^, we use here the largest connected component of the aggregated free Skype service as the underlying structure, where nodes are Skype users and links confirmed contacts between them. This is a good approximation since it maps all connections in the Skype social network without sampling, and the paid service is only available for individuals already enrolled in the Skype network. Also note that the service adoption process evolves in a considerably faster time-scale than the underpinning social network. This way applying a time-scale separation, and considering the network to be static, may provide a good first approximation here. The underlying structure is an aggregate from September 2003 to November 2011 (i.e. over 99 months) and contains roughly 4.4 billion links and 510 million registered users worldwide^49^. The data is fully anonymised and considers only confirmed connections between users (for more data details see SI).

In what follows we first provide empirical evidence of the distribution of individual adoption thresholds and other structural and dynamical features of a worldwide adoption cluster. We incorporate the observed structural and threshold heterogeneities into a dynamical threshold model where multiple nodes adopting spontaneously (i.e. firstly among their neighbours) are allowed^50^. We find that if the fraction of users who reject to adopt the product is large, the system enters a quenched state where the evolution and structure of the global adoption cluster is very similar to our empirical observations. Model calculations and the analysis of the real social contagion process suggest that the evolving structure of an adoption cluster differs radically from what has been proposed earlier^9^, since it is triggered by several spontaneous adoptions arriving at a constant rate, while stable adopters who are initially resisting exposure, are actually responsible for the emergence of global social adoption (Fig. 1b,c).

## Results

Social contagion phenomena can be modelled as binary-state processes evolving on networks and driven by threshold mechanisms. In these systems individuals are represented by nodes, each being either in a susceptible (0) or adopter (1) state and influencing each other by transferring information via social ties^6^. Nodes are connected in a network with degree distribution *P*(*k*) and average degree *z* = 〈*k*〉. In addition, each node has an individual threshold *ϕ* ∈ [0, 1] drawn from a distribution *P*(*ϕ*) with average *w* = 〈*ϕ*〉. This threshold determines the minimum fraction of exposed neighbours that triggers adoption and captures the resistance of an individual against engaging in spreading behaviour. Once a node reaches its threshold, it switches state from 0 to 1 and keeps it until the end of the dynamics. In his seminal paper about threshold dynamics, Watts^9^ classified nodes into three categories based on their threshold and degree. He identified *innovator* nodes that spontaneously change state to 1, thus starting the process. Such nodes have a trivial threshold *ϕ* = 0. Then there are nodes with threshold 0 < *ϕ* ≤ 1/*k*, called *vulnerable*, which need one adopting neighbour before their own adoption. Finally, there are more resilient nodes with threshold *ϕ* > 1/*k*, denoted as *stable*, referring to individuals in need of strong social influence to follow the actions of their acquaintances.

In the WT model^9^, small perturbations (like the spontaneous adoption of a single seed node) can trigger global cascading patterns. However, their emergence is subject to the so-called *cascade condition*: the innovator seed has to be linked to a percolating vulnerable cluster, which adopts immediately afterwards and further triggers a global cascade (i.e. a set of adopters larger than a fixed fraction of the finite network). The cascade condition is satisfied if the network is inside a bounded regime in (*w*, *z*)-space^9^. This regime depends on degree and threshold heterogeneities^9^ and may change its shape if several innovators start the process^42^. In addition, while models with more sophisticated social influence function can be introduced^24^,^51^ the original linear-threshold assumption proposed by Watts and Granovetter seems to be sufficient to interpret our observations.

### Empirical observations

**Figure 1:**
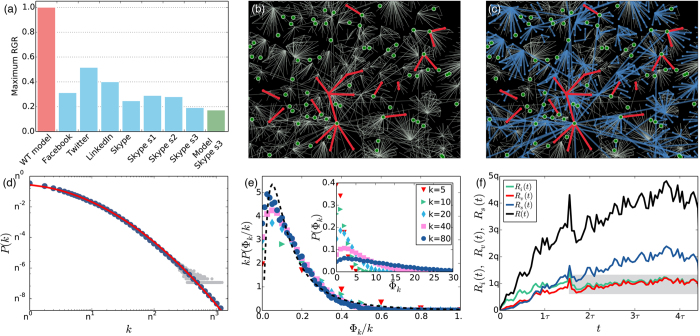
Structure and dynamics of online service adoption. (**a**) Yearly maximum relative growth rate (RGR) of cumulative adoption (see Appendix) for several online social-communication services^48^, including three Skype paid services (s1 - “subscription”, s2 - “voicemail”, and s3 - “buy credit”). The red bar corresponds to a rapid cascade of adoption suggested by the Watts threshold (WT) model, while the green bar is the model prediction for Skype s3. (**b,c**) Snowball sample of the Skype social network (gray links) with nodes and links coloured according to their adoption state: multiple innovators (green nodes), induced small vulnerable trees (red nodes and links), and the triggered connected stable cluster (blue nodes and links). Note that some vulnerable and stable clusters seemingly appear without an innovator seed due to the finite distance used in the snowball sampling method. (**d**) Degree distribution *P*(*k*) of the Skype network (gray/blue circles for raw/binned data) on double log-scale with arbitrary base *n. P*(*k*) is fitted by a lognormal distribution (see Appendix and SI) with parameters *μ*_*D*_ = 1.2 and *σ*_*D*_ = 1.39, and average *z* = 8.56 (red line). (**e**) Distribution *P*(Φ_*k*_) of integer thresholds Φ_*k*_ for several degree groups in Skype s3 (inset). By using *P*(Φ_*k*_, *k*) = *kP*(Φ_*k*_/*k*), these curves collapse to a master curve approximated by a lognormal function (dashed line in main panel) with parameters *μ*_*T*_ = −2 and *σ*_*T*_ = 1, as constrained by the average threshold *w* = 0.19 (see Appendix and SI). (**f**) Adoption rate of innovators [*R*_*i*_(*t*)], vulnerable nodes [*R*_*v*_(*t*)], and stable nodes [*R*_*s*_(*t*)], as well as net service adoption rate [*R*(*t*)]. Rates are measured with a 1-month time window, while *q* and *τ* are arbitrary constants. The shaded area indicates the regime where innovators adopt approximately with constant rate.

Degree and threshold heterogeneities are indeed present in the social network of Skype. The degree distribution *P*(*k*) is well approximated by a lognormal function 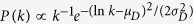 (*k* ≥ *k*_min_) with parameters *μ*_*D*_ = 1.2, *σ*_*D*_ = 1.39 and *k*_min_ = 1 (Fig. 1d), giving an average degree *z* = 8.56 (for goodness of fit see SI). Moreover, at the time of adoption we can measure the threshold *ϕ* = Φ_*k*_/*k* of a user by counting the number Φ_*k*_ of its neighbours who have adopted the service earlier. We then group users by degree and calculate the distribution *P*(Φ_*k*_) of the integer threshold Φ_*k*_^38^ (Fig. 1e). By using the scaling relation *P*(Φ_*k*_, *k*) = *kP*(Φ_*k*_/*k*) all distributions collapse to a master curve well approximated by a lognormal function 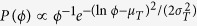, with parameters *μ*_*T*_ = −2 and *σ*_*T*_ = 1 as constrained by the average threshold *w* = 0.19 (see Appendix and SI). Note that we observe qualitatively the same scaling and lognormal shape of the threshold distribution for another service (see SI). These empirical observations, in addition to the broad degree distribution, provide quantitative evidence about the heterogeneous nature of adoption thresholds.

Since we know the complete structure of the online social network, as well as the first time of service usage for all adopters, we can follow the temporal evolution of the adoption dynamics. By counting the number of adopting neighbours of an ego, we identify innovators (Φ_*k*_ = 0), and vulnerable (Φ_*k*_ = 1) or stable (Φ_*k*_ > 1) nodes. The adoption rates for these categories behave rather differently from previous suggestions^9^ (Fig. 1f). First, there is not only one seed but an increasing fraction of innovators in the system who, after an initial period, adopt approximately at a constant rate. Second, vulnerable nodes adopt approximately with the same rate as innovators suggesting a strong correlation between these types of adoption. This stationary behaviour is rather surprising as environmental effects, like competition or marketing campaigns, potentially influence the adoption dynamics. Nevertheless, this pattern appears to be consistent among the two investigated services (also see Fig. S5 in SI), where innovator and vulnerable adoptions evolve with approximately constant rates beyond statistical and periodic (circadian, monthly, yearly) fluctuations. On the other hand, the overall adoption process accelerates due to the increasing rate of stable adoptions induced by social influence. At the same time a giant adoption cluster grows and percolates through the whole network (Fig. 3a, main panel). Despite of this expansion dynamics and connected structure of the service adoption cluster, the service reaches less than 6% of the total number of active Skype users over a period of 7 years^49^. Therefore we ask whether one can refer to these adoption clusters as cascades. They are not triggered by a small perturbation but induced by several innovators; their evolution is not instantaneous but ranges through several years; and although they involve millions of individuals, they reach only a reduced fraction of the whole network. To answer we incorporate the above mentioned features into a dynamical threshold model^50^ with a growing group of innovators and investigate their effect on the evolution of global social adoption. Note that although we cannot follow the direct pathways of social influence, we perform a null model study to demonstrate at the system level that social influence is present and dominates the contagion process, as compared to effects of homophily (see section S3 of the SI, together with another empirical spreading scenario in S7.1).

### Model

Our modelling framework is an extension to conventional threshold dynamics on networks studied by Watts, Gleeson, Singh, and others, where all nodes are initially susceptible and innovators are only introduced as an initial seed of arbitrary size^9^,^15^,^42^,^43^. Apart from the threshold rule discussed above, our model considers two additional features: (i) a fraction *r* of ‘immune’ nodes that never adopt, indicating lack of interest in the service; (ii) due to external influence, susceptible nodes adopt the innovation spontaneously (i.e. become innovators) throughout time with constant rate *p*_*n*_, rather than only at the beginning of the dynamics. In this way, the dynamical evolution of the system is completely defined by the online social network, the distribution *P*(*ϕ*) and the parameters *r*, *p*_*n*_. For the sake of simplicity we consider a configuration-model network, i.e., we ignore correlations in the social network and characterise it solely by its degree distribution *P*(*k*). Furthermore, node degrees and thresholds are considered to be independent^38^,^52^,^53^. We remark that somewhat similar concepts called “stubborn nodes” mimicking individuals’ resistance against adoption^54^,^55^, and “global nodes” capturing adoptions driven by external effects^56^ have been considered in threshold models with a rich variety of effects on cascading behaviour.

Our threshold model, which has also been introduced in^50^, can be studied analytically by extending the framework of approximate master equations (AMEs) for monotone binary-state dynamics recently developed by Gleeson^38^,^52^,^53^, where the transition rate between susceptible and adoption states only depends on the number *m* of network neighbours that have already adopted. We describe a node by the property vector **k** = (*k*, *c*), where *k* = *k*_0_, *k*_1_, …*k*_*M*−1_ is its degree and *c* = 0, 1, …, *M* its type, i.e. *c* = 0 is the type of the fraction *r* of immune nodes, while *c* ≠ 0 is the type of all non-immune nodes that have threshold *ϕ*_*c*_. In this way *P*(*ϕ*) is substituted by the discrete distribution of types *P*(*c*) (for *c* > 0). The integer *M* is the maximum number of degrees (or non-zero types) considered in the AME framework, which can be increased to improve the accuracy of the analytical approximation at the expense of speed in its numerical computation (see S4.2). Under these conditions, the AME system describing the dynamics of the threshold model is reduced to the pair of ordinary differential equations (see SI),


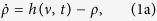



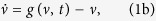


where *ρ*(*t*) is the fraction of adopters in the network, *ν*(*t*) is the probability that a randomly chosen neighbour of a susceptible node is an adopter, and the initial conditions are *ρ*(0) = *ν*(0) = 0. Here,


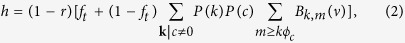


and,


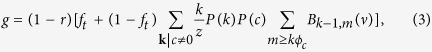


where 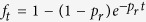, *p*_*r*_ = *p*_*n*_/(1 − *r*), and 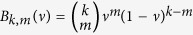 is the binomial distribution. The fraction of adopters *ρ* is then obtained by solving Eq. (1) numerically. Since susceptible nodes adopt spontaneously with rate *p*_*n*_, the fraction of innovators *ρ*_0_(*t*) in the network is given by (see S4.3),


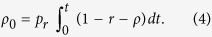


**Figure 2:**
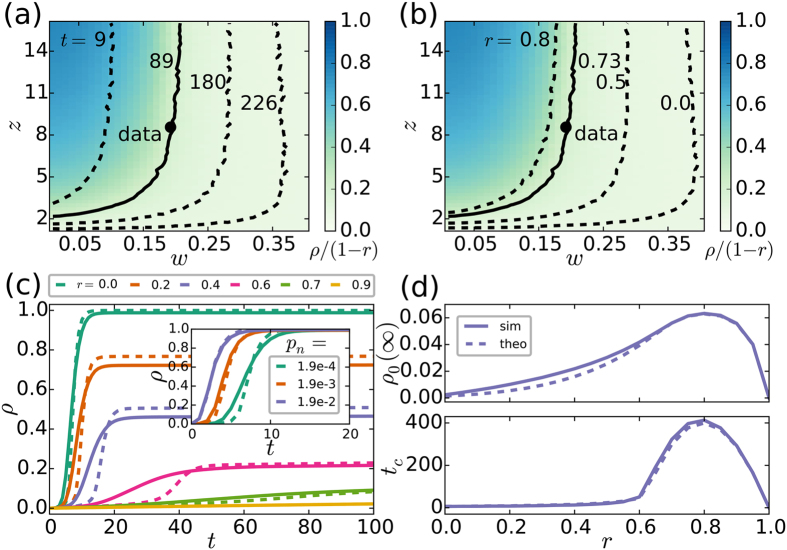
Threshold model for the adoption of online services. **(a,b)** Surface plot of the normalised fraction of adopters *ρ*/(1 − *r*) in (*w*, *z*)-space, for *r* = 0.73 and *t* = 89. Contour lines signal parameter values for which 20% of non-immune nodes have adopted, for fixed *r* and varying time (**a**), and for fixed time and varying *r*. (**b**) The continuous contour line and dot indicate parameter values in the last observation of Skype s3. A regime of maximal adoption (*ρ* ≈ 1 − *r*) grows as time goes by, and shrinks for larger *r*. **(c)** Time series of the fraction of adopters *ρ* for fixed *p*_*n*_ = 0.00019 and varying *r* (main), and for fixed *r* = 0 and varying *p*_*n*_ (inset). These curves are well approximated by the solution of Eq. (1) for *k*_0_ = 3, *k*_*M*−1_ = 150 and *M* = 25 (dashed lines). The dynamics is clearly faster for larger *p*_*n*_. As *r* increases, the system enters a regime where the dynamics is slowed down and adopters are mostly innovators. **(d)** Final fraction of innovators *ρ*_0_(∞) and time *t*_*c*_ when 50% of non-immune nodes have adopted as a function of *r*, both simulated and theoretical. The crossover to a regime of slow adoption is characterised by a maximal fraction of innovators and time *t*_*c*_. Unless otherwise stated, *p*_*n*_ = 0.00019 and we use *N* = 10^4^, *μ*_*D*_ = 1.09, *σ*_*D*_ = 1.39, *k*_min_ = 1, *μ*_*T*_ = −2, and *σ*_*T*_ = 1 to obtain *z* = 8.56 and *w* = 0.19 as in Skype s3. The difference in *μ*_*D*_ between data and model is due to finite-size effects (see Appendix). Numerical results are averages over 10^2^ (a-b) and 10^3^ (c-d) realisations.

We also implement the threshold model numerically via a Monte Carlo simulation in a network of size *N*, with a lognormal degree distribution and a lognormal threshold distribution as observed empirically. Thus, we can explore the behaviour of *ρ* and *ρ*_0_ as a function of *z*, *w*, *p*_*n*_ and *r*, both in the numerical simulation and in the theoretical approximation given by Eqs (1) and (4). For *p*_*n*_ > 0 some nodes adopt spontaneously as time passes by, leading to a frozen state characterised by a final fraction *ρ*(∞) = 1 − *r* of adopters. However, the time needed to reach such state depends heavily on the distribution of degrees and thresholds, as signalled by a region of large adoption (*ρ* ≈ 1 − *r*) that grows in (*w*, *z*)-space with time (contour lines in Fig. 2a). If we fix a time in the dynamics and vary the fraction of immune nodes instead, this region shrinks as *r* increases (contour lines in Fig. 2b). In other words, the set of networks (defined by their average degree and threshold) that allow the spread of adoption is larger at later times in the dynamics, or when the fraction of immune nodes is small. When both *t* and *r* are fixed, the normalised fraction of adopters *ρ*/(1 − *r*) gradually decreases for less connected networks with larger thresholds (surface plot in Fig. 2a,b).

For *r* ≈ 0 the critical fraction of innovators necessary to trigger a cascade of fast adoption throughout all susceptible nodes may be identified as the inflection point in the time series of *ρ* (Fig. 2c, inset). The adoption cascade appears sooner for larger *p*_*n*_, since this parameter regulates how quickly the critical fraction of innovators is reached. Yet as we increase *r* above a threshold *r*_*c*_, the system enters a regime where rapid cascades disappear and adoption is slowed down. The crossover between these regimes is gradual, as seen in the shape of *ρ* for increasing *r* (Fig. 2c, main panel). We may identify *r*_*c*_ in various ways: by the maximum in both the final fraction of innovators *ρ*_0_(∞) and the critical time *t*_*c*_ when *ρ* = (1 − *r*)/2 (Fig. 2d), or as the *r* value where the inflection point in *ρ* disappears. These measures indicate *r*_*c*_ ≈ 0.8 for the chosen parameters. All global properties of the dynamics (like the functional dependence of *ρ* and *ρ*_0_) are very well approximated by the solution of Eqs (1) and (4) (dashed lines in Fig. 2c,d). Indeed, the AME framework is able to capture the shape of the *ρ* time series, the crossover between regimes of fast and slow adoption, as well as the maximum in *ρ*_0_(∞) and *t*_*c*_.

### Validation

**Figure 3:**
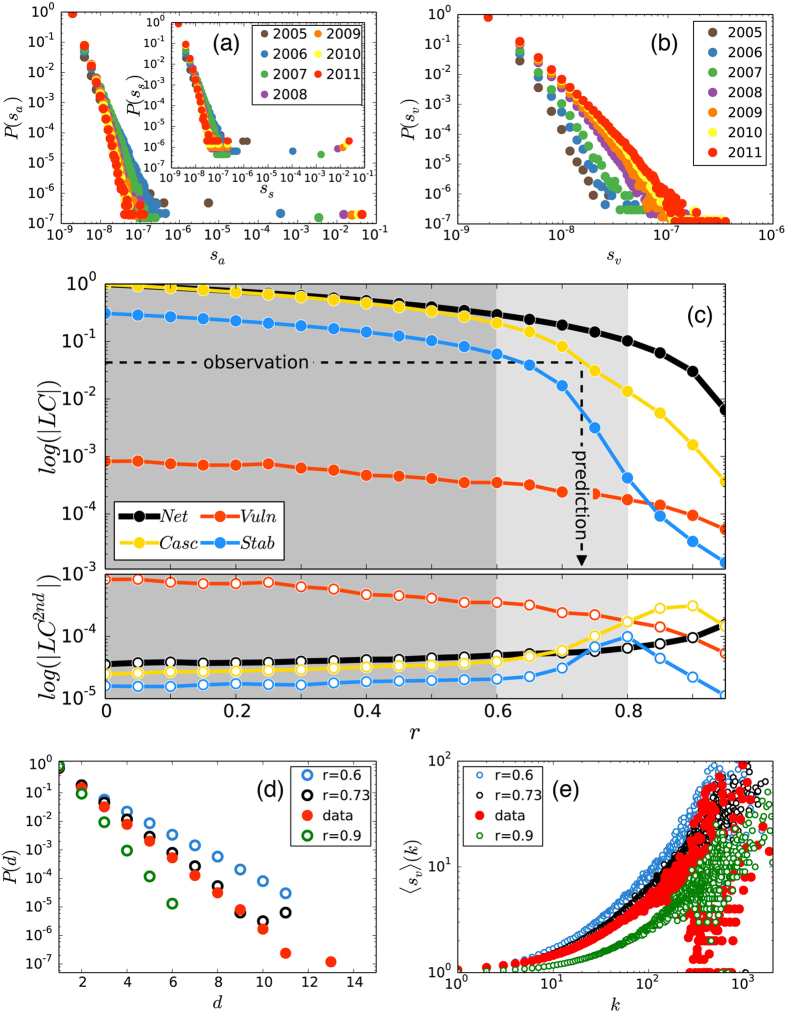
Empirical cluster statistics and simulation results. (**a**) Empirical connected-component size distribution at different times for the adoption [*P*(*s*_*a*_), main panel] and stable adoption [*P*(*s*_*s*_), inset] networks, with *s*_*a*_ and *s*_*s*_ relative to system size. (**b**) Empirical connected-component size distribution *P*(*s*_*v*_) for the relative size of innovator-induced vulnerable trees at different times. (**c**) Average size of the largest (*LC*) and 2nd largest (*LC*^2*nd*^) components of the model network (‘Net’), adoption network (‘Casc’), stable network (‘Stab’), and induced vulnerable trees (‘Vuln’) as a function of *r*. Dashed lines show the observed relative size of the real *LC* of the adopter network in 2011 [see main panel in (**a**)] and the predicted *r* value. (**d**) Distribution *P*(*d*) of depths of induced vulnerable trees in both data and model for several *r* values, showing a good fit with the data for *r* = 0.73. The difference in the tail is due to finite-size effects. (**e**) Correlation 〈*s*_*v*_〉(*k*) between innovator degree and average size of vulnerable trees in both data and model with the same *r* values as in (**e**). Model calculations for (**d,e**) correspond to networks of size *N* = 10^6^ and are averaged over 10^2^ realisations.

To better understand how innovation spreads throughout real social networks, we take a closer look at the internal structure of the service adoption process. By taking into account individual adoption times we construct an evolving adoption network with links between users who have adopted the service before time *t* and are connected in the social structure. In order to avoid the effect of instantaneous group adoptions (evidently not driven by social influence), we only consider links between nodes who are neighbours in the underlying social network and whose adoption did not happen at the same time. This way links in the adoption graph indicate ties where social influence among individuals could have existed. The size distribution *P*(*s*_*a*_) of connected components in the adoption network shows the emergence of a giant percolating component over time (Fig. 3a), along with several other small clusters. Moreover, after decomposition we observe that the giant cluster does not consist of a single innovator seed and percolating vulnerable tree^9^, but builds up from several innovator seeds that induce small vulnerable trees locally (Fig. 3b), each with small depth (Fig. 3d)^34^,^57^. At the same time the stable adoption network (considering connections between all stable adopters at the time) has a giant connected component, indicating the emergence of a percolating stable cluster with size comparable to the largest adoption cluster (Fig. 3a, inset). These observations suggest a scenario for the evolution of the global adoption component different from earlier threshold models^9^. It appears that here multiple innovators adopt at different times and trigger local vulnerable trees (Fig. 1b), which in turn induce a percolating component of connected stable nodes that holds the global adoption cluster together (Fig. 1c). Consequently, in the structure of the adoption network primary triggering effects are important only locally, while external and secondary triggering mechanisms seem to be responsible for the emergence of global-scale adoption.

To model the observed dynamics and explore the effect of immune nodes, we perform extensive numerical simulations of the threshold model with parameters determined directly from the data (see Appendix and SI). We use a network structure with empirical degree and threshold distributions and fix *p*_*n*_ = 0.00019 as the constant rate of innovators, implying that the time scale of a Monte Carlo iteration in the model is 1 month. We measure the average size of the largest (*LC*) and second largest (*LC*^2*nd*^) connected components of the background social network, and of the stable, vulnerable and global adoption networks, as a function of the fraction of immune nodes *r*. After *T* = 89 iterations (matching the length of the real observation period) we identify three regimes of the dynamics (Fig. 3c): if 0 < *r* < 0.6 (dark-shaded area) the spreading process is very rapid and evolves in a global cascade, which reaches most of the nodes of the shrinking susceptible network in a few iteration steps. About 10% of adopters are connected in a percolating stable cluster, while vulnerable components remain very small in accordance with empirical observations. In the crossover regime 0.6 < *r* < 0.8 (light-shaded area), the adoption process slows down considerably (Fig. 2d, lower panel), as stable adoptions become less likely due to the quenching effect of immune nodes. The adoption process becomes the slowest at *r*_*c*_ = 0.8 (Fig. 2d, lower panel) when the percolating stable cluster falls apart, as demonstrated by a peak in the corresponding *LC*^2*nd*^ curve (Fig. 3c, lower panel). Finally, around *r* = 0.9 the adoption network becomes fragmented and no global diffusion takes place. We repeat the same calculations for another service and find qualitatively the same picture, but with the crossover regime shifted towards larger *r* values due to the different parametrisation of the model process. Note that another possible reason for the slow adoption could be the time users wait between their threshold has been reached and actual adoption. We test for the effect of this potential scenario on the empirical curves but find no qualitative change in the dynamics (see SI).

We can use these calculations to estimate the only unknown parameter (the fraction *r* of immune nodes in Skype) by matching the size of the largest component (*LC*_*Net*_) between real and model adoption networks at time *T*. Empirically, this value is the relative size corresponding to the last point on the right-hand side of the distribution for 2011 (Fig. 3a, main panel). The corresponding value in the model is *r* = 0.73 (dashed lines in Fig. 3c; also Fig. 2a,b), suggesting that the real adoption process lies in the crossover regime. The other analysed service turns out to lie right of the crossover regime, which explains its large innovator adoption rate and reduced size of stable and vulnerable adoption clusters (see SI).

To test the validity of the prediction of *r* we perform three different calculations. First we measure the maximum relative growth rate of cumulative adoption and find a good match between model and data (Skype s3 and Model Skype s3 in Fig. 1a). In other words, the model correctly estimates the speed of the adoption process. Second, we measure the distribution *P*(*d*) of depths of induced vulnerable trees (Fig. 3d). Finally, in order to verify earlier theoretical suggestions^42^, we look at the correlation 〈*s*_*v*_〉(*k*) between the degree of innovators and the average size of vulnerable trees induced by them (Fig. 3e). We perform the last two measurements on the real data and in the model process for *r* = 0.6 and 0.9, as well as for the predicted value *r* = 0.73. In the case of 〈*s*_*v*_〉(*k*), we find a strong positive correlation in the data, explained partially by degree heterogeneities in the underlying social network, but surprisingly well emulated by the model. More importantly, although both quantities appear to scale with *r*, measures for the estimated *r* value fit the empirical data remarkably well, confirming our earlier validation based on the matching of relative component sizes (for further discussion see SI).

## Discussion

Although some products and innovations diffusing in society may cover a large fraction of the population, their spreading tends to follow slow cascading patterns, the dynamics of which have been modelled before by simple diffusion models like that of Bass^18^. However, this approach neglects threshold mechanisms that arguably drive the decision making of single individuals. On the other hand, threshold models study the conditions for cascades in global diffusion but do not address their temporal evolution, which is clearly a relevant factor in real-world adoption processes. These models are commonly used to predict rapid cascading patterns of adoption, which is a more realistic scenario for the spreading of information, opinions, or behavioural patterns but are not observed in the case of product or innovation diffusion where adoption requires additional efforts, e.g., free or paid registration. Here we provide a solution for this conundrum by analysing and modelling the worldwide spread of an online service in the techno-social communication network of Skype. Beyond the novel empirical evidence about heterogeneous adoption thresholds and non-linear dynamics of the adoption process, we identify two additional components necessary to introduce in the modelling of product adoption, namely: (a) a constant flow of innovators, which may induce rapid adoption cascades even if the system is initially out of the cascading regime; and (b) a fraction of immune nodes that forces the system into a quenched state where adoption slows down. These features are responsible for a critical structure of empirical adoption components that radically differs from previous theoretical expectations. We incorporate these mechanisms into a threshold model controlled by the rate of innovators and the fraction of immune nodes. The model contains several simplifying assumptions, e.g., about the functional form of social influence, the uniformity of nodes with the same degree, or about ignoring homophily in the adoption process, however, it is able to reproduce several pathways ranging from cascading behaviour to more realistic dynamics of innovation adoption. By constraining the model with empirically determined parameters, we provide an estimate for the real fraction of susceptible agents in the social network of Skype, and validate this prediction through correlated structural features matching empirical observations.

Our aim in this study was to provide empirical observations as well as methods and tools to model the dynamics of social contagion phenomena with the hope it will foster thoughts for future research. One possible direction would be the observation of the reported structure and evolution of the global adoption cluster in other systems similar to the ones studied in^26^,^28^,^29^,^34^,^36^,^57^. Other promising directions could be the consideration of homophilic or assortative structural correlations, or the evolving nature of the underpinning social network with timely created and dissolved social ties (as studied in^21^), or the effects of interpersonal influence or leader-follower mechanisms on the social contagion process. Finally, we hope that the reported results may improve efficiency in the strategies of enhancing the diffusion of products and innovations, by shifting attention from the creation of short-lived perturbations to the sustenance of external input.

## Material and Methods

### Data description

We use a static representation of the Skype social network aggregated over 99 months between September 2003 and November 2011. We follow the adoption of the “buy credit” paid service for 89 months starting from 2004, and the paid service “subscription” for 42 months starting from 2008 (for further details about the network and service see SI). By considering the online social structure and adoption times, we identify users as innovator, vulnerable, or stable nodes based on the number Φ_*k*_ of adopting neighbours at the time of exposure. Thresholds are calculated as *ϕ* = Φ_*k*_/*k* for users with *k* contacts. The adoption network is constructed by considering confirmed social links between users who adopted the service earlier than *t*. In order to avoid the effect of instantaneous group adoptions (evidently not driven by social influence), we only consider links between nodes who are neighbours in the underlying social network and whose adoption did not happen at the same time. Note that for the categorisation of nodes we use only the adoption time and the state of their peers, and thus real categories may differ slightly. For example, an innovator may appear as a vulnerable or stable node, even if its decision was not driven by social influence but some of its peers adopted earlier. To consider this bias we measure effective rates of adoption for the model process as well, just like for the empirical case (Fig. 1) and section S3.

### Maximum relative growth rate

This measure is obtained by taking the maximum of the yearly adoption rate (yearly count of adoptions) normalised by the final observed adoption number of a given service. It characterises the maximum speed of adoption a service experienced during its history and takes values between 0 (no cascade) and 1 (instantaneous cascade). We repeat this measurement for the estimated number of registered users of Facebook, Twitter, and LinkedIn^48^, as well as for the number of active users of Skype and three paid Skype services. Adoption rates for Facebook, Twitter, and LinkedIn correspond to the period between 2006 and 2012, and for Skype and its services to the interval from release date until 2011.

### Empirical parameter estimation

We use the Skype data to directly determine all model parameters, apart from the fraction *r* of immune nodes. To best approximate the degree distribution of the real network, after testing different candidate functions (see SI) we select a lognormal function 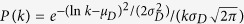 with parameters *μ*_*D*_ = 1.2 and *σ*_*D*_ = 1.39 and minimum degree *k*_min_ = 1, leading to the average degree *z* = 8.56. To account for finite-size effects in the model results for low *N* (Fig. 2), we decrease *μ*_*D*_ slightly to obtain the same value of *z* as in the real network.

The threshold distribution of each degree group collapses to a master curve after normalisation by using the scaling relation *P*(Φ_*k*_, *k*) = *kP*(Φ_*k*_/*k*). This master curve can be well approximated by the lognormal distribution 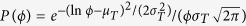, with parameters *μ*_*T*_ = −2 and *σ*_*T*_ = 1 as determined by the empirical average threshold *w* = 0.19 and standard deviation 0.233 (for further details see SI). We estimate a rate of innovators *p*_*n*_ = 0.00019 by fitting a constant function to *R*_*i*_(*t*) for *t* > 2*τ* (Fig. 1f). The fit to *p*_*n*_ also matches the time scale of a Monte Carlo iteration in the model to 1 month. Model results (Fig. 3d,e) are calculated with *r* = 0.73 and *p*_*n*_ = 0.00019. Simulation results in Fig. 3c–e are averaged over 100 configuration-model networks of size *N* = 10^5^ (10^6^) after *T* = 89 iterations, matching the length of the observation period in Skype.

### Model description

We characterise the static social network by the extended distribution *P*(**k**), where *P*(**k**) = *rP*(*k*) for *c* = 0 and *P*(**k**) = (1 − *r*)*P*(*k*)*P*(*c*) for *c* > 0. Non-immune, susceptible nodes with property vector **k** adopt spontaneously at a constant rate *p*_*n*_, else they adopt only if a fraction *ϕ*_*c*_ of their *k* neighbours have adopted before. These rules are condensed in the probability *F*_**k**,*m*_*dt* that a node will adopt in a small time interval *dt*, given that *m* of its neighbours are already adopters,


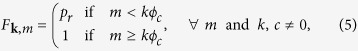


with *F*_(*k*,0),*m*_ = 0 ∀*k*, *m* and *F*_(0,*c*),0_ = *p*_*r*_ ∀*c* ≠ 0 (for immune and isolated nodes, respectively). The dynamics of adoption is well described by an AME for the fraction *s*_**k**,*m*_(*t*) of **k**-nodes that are susceptible at time *t* and have *m* = 0, …, *k* adopting neighbours^52^,^53^,^58^,


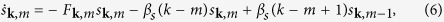


where 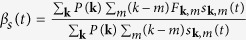. To reduce the dimensionality of Eq. (6) we consider the ansatz 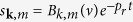 for *m* < *kϕ*_*c*_, leading to the condition 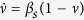. With 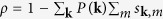 and some algebra, this condition is reduced to Eq. (1) (see SI).

## Additional Information

**How to cite this article**: Karsai, M. *et al.* Local cascades induced global contagion: How heterogeneous thresholds, exogenous effects, and unconcerned behaviour govern online adoption spreading. *Sci. Rep.* **6**, 27178; doi: 10.1038/srep27178 (2016).

## Supplementary Material

Supplementary Information

## References

[b1] Centola D. The spread of behavior in an online social network experiment. Science 329, 1194–1197 (2010). 20813952 10.1126/science.1185231

[b2] McPherson M., Smith-Lovin L. & Cook J. M. Birds of a Feather: Homophily in Social Networks. Ann. Rev. Sociol. 27, 415–444 (2001).

[b3] Toole J. L., Cha M. & González M. C. Modeling the adoption of innovations in the presence of geographic and media influences. PLoS ONE 7, e29528 (2012). 22276119 10.1371/journal.pone.0029528 PMC3261844

[b4] Castellano C., Fortunato S. & Loreto V. Statistical physics of social dynamics. Rev. Mod. Phys. 81, 591–646 (2009).

[b5] Rogers E. M. Diffusion of Innovations. (Simon & Schuster), 5th edition (2003).

[b6] Granovetter M. Threshold models of collective behavior. Am. J. Sociol. 83, 1420–1443 (1978).

[b7] Schelling T. C. Models of segregation. Am. Econ. Rev. 59, 488–493 (1969).

[b8] Axelrod R. The dissemination of culture. J. Conflict Resolut. 41, 203–226 (1997).

[b9] Watts D. J. A simple model of global cascades on random networks. Proc. Natl. Acad. Sci. USA 99, 5766–5771 (2002). 16578874 10.1073/pnas.082090499 PMC122850

[b10] Handjani S. Survival of threshold contact processes. J. Theo. Probab. 10, 737–746 (1997).

[b11] Valente T. W. Social network thresholds in the diffusion of innovations. Social Networks 18, 69–89 (1996).

[b12] Watts D. J. & Dodds P. S. Influentials, networks, and public opinion formation. J. Consum. Res. 34, 441–458 (2007).

[b13] Melnik S., Ward J. A., Gleeson J. P. & Porter M. A. Multi-stage complex contagions. Chaos 23, 013124 (2013). 23556961 10.1063/1.4790836

[b14] Gómez V., Kappen H. J. & Kaltenbrunner A. Modeling the structure and evolution of discussion cascades. (HT’11, ACM, New York, NY, USA), pp. 181–190 (2010).

[b15] Karampourniotis P. D., Sreenivasan S., Szymanski B. K. & Korniss Gy. The Impact of Heterogeneous Thresholds on Social Contagion with Multiple Initiators. PLoS ONE 10(11), e0143020 (2015). 26571486 10.1371/journal.pone.0143020 PMC4646465

[b16] Centola D. & Macy M. Complex contagions and the weakness of long ties. Am. J. Sociol. 113, 702–734 (2007).

[b17] Barrat A., Barthélemy M. & Vespignani V. Dynamical Processes on Complex Networks. (Cambridge University Press, 2008).

[b18] Bass F. M. A new product growth for model consumer durables. Manage. Sci. 15, 215–227 (1969).

[b19] Aral S., Muchnika L. & Sundararajana A. Distinguishing influence-based contagion from homophily-driven diffusion in dynamic networks. Proc. Natl. Acad. Sci. USA 106, 21544–21549 (2009). 20007780 10.1073/pnas.0908800106 PMC2799846

[b20] Shalizi C. R. & Thomas A. C. Homophily and Contagion Are Generically Confounded in Observational Social Network Studies. Sociol Methods Res. 40, 211–239 (2011). 22523436 10.1177/0049124111404820 PMC3328971

[b21] Karsai M., Iñiguez G., Kaski K. & Kertész J. Complex contagion process in spreading of online innovation. J. Roy. Soc. Interface 11, 20140694 (2014). 25339685 10.1098/rsif.2014.0694 PMC4223898

[b22] Holt C. A. Markets, Games, Strategic Behavior. (Addison Wesley, 2006).

[b23] Bikhchandani S., Hirshleifer D. & Welch I. A theory of fads, fashion, custom, and cultural change as informational cascades. J. Polit. Econ. 100, 992–1026 (1992).

[b24] Latané B. The psychology of social impact. Am. Psycholog. 36 4, 343–356 (1981).

[b25] González-Bailón S., Borge-Holthoefer J., Rivero A. & Moreno Y. The dynamics of protest recruitment through an online network. Sci. Rep. 1, 197 (2011). 22355712 10.1038/srep00197 PMC3240992

[b26] Borge-Holthoefer J. *et al.* Structural and dynamical patterns on online social networks: The Spanish May 15th movement as a case study. PLoS ONE 6, e23883 (2011). 21886834 10.1371/journal.pone.0023883 PMC3158778

[b27] Ellis C. J. & Fender J. Information cascades and revolutionary regime transitions. Econ. J. 121, 763–792 (2011).

[b28] Dow P. A., Adamic L. A. & Friggeri A. The anatomy of large Facebook cascades. (ICWSM, AAAI, Boston, MA, USA), pp. 145–154 (2013).

[b29] Gruhl D., Guha R., Nowell D. L. & Tomkins A. Information diffusion through blogspace. (WWW ‘04, ACM, New York, NY, USA), pp. 491-501 (2004).

[b30] Baños R. A., Borge-Holthoefer J. & Moreno Y. The role of hidden influentials in the diffusion of online information cascades. EPJ Data Sci. 2, 6 (2013).

[b31] Hale H. E. Regime change cascades: What we have learned from the 1848 revolutions to the 2011 Arab uprisings. Annu. Rev. Polit. Sci. 16, 331–353 (2013).

[b32] Leskovec J., Singh A. & Kleinberg J. Patterns of influence in a recommendation network. (PAKDD ‘06, Singapore), pp. 380–389 (2006).

[b33] Leskovec J., Adamic L. A. & Huberman B. A. The dynamics of viral marketing. (TWEB, ACM, New York, NY, USA), vol. 1, pp. 5 (2007).

[b34] Goel S., Watts D. J. & Goldstein D. G. The structure of online diffusion networks. (EC ‘12, ACM, New York, NY, USA), pp. 623–638 (2012).

[b35] Fowler J. H. & Christakis N. A. Cooperative behavior cascades in human social networks. Proc. Natl. Acad. Sci. USA 107, 5334–5338 (2009). 20212120 10.1073/pnas.0913149107 PMC2851803

[b36] Borge-Holthoefer J., Baños R. A., González-Bailón S. & Moreno Y. Cascading behaviour in complex socio-technical networks. J. Complex Net. 1, 1–22 (2013).

[b37] Hackett A. & Gleeson J. P. Cascades on clique-based graphs. Phys. Rev. E 87, 062801 (2013). 10.1103/PhysRevE.87.062801 23848723

[b38] Gleeson J. P. Cascades on correlated and modular random networks. Phys. Rev. E 77, 046117 (2008). 10.1103/PhysRevE.77.046117 18517700

[b39] Brummitt C. D., D’Souza R. M. & Leicht E. A. Suppressing cascades of load in interdependent networks. Proc. Natl. Acad. Sci. USA 109, E680–E689 (2011). 22355144 10.1073/pnas.1110586109 PMC3311366

[b40] Ghosh R. & Lerman K. A framework for quantitative analysis of cascades on networks, WSDM ‘11. (WSDM ‘11, ACM, New York, NY, USA), pp. 665–674 (2010).

[b41] Hurd T. R. & Gleeson J. P. On Watts’ cascade model with random link weights. J. Complex Net. 1, 25–43 (2013).

[b42] Singh P., Sreenivasan S., Szymanski B. K. & Korniss Gy. Threshold-limited spreading in social networks with multiple initiators. Sci. Rep. 3, 2330 (2013). 23900230 10.1038/srep02330 PMC3728590

[b43] Gleeson J. P. & Cahalane D. J. Seed size strongly affects cascades on random networks. Phys. Rev. E 75, 050101(R) (2007). 10.1103/PhysRevE.75.056103 17677129

[b44] Yağan O. & Gligor V. Analysis of complex contagions in random multiplex networks. Phys. Rev. E 86, 036103 (2012). 10.1103/PhysRevE.86.036103 23030976

[b45] Brummitt C. D. & Kobayashi T. Cascades in multiplex financial networks with debts of different seniority. Phys. Rev. E 91, 062813 (2015). 10.1103/PhysRevE.91.062813 26172760

[b46] Karimi F. & Holme P. Threshold model of cascades in empirical temporal networks. Physica A 392, 16 (2013).

[b47] Backlund V.-P., Saramäki J. & Pan R. K. Effects of temporal correlations on cascades: Threshold models on temporal networks. Phys. Rev. E 89, 062815 (2014). 10.1103/PhysRevE.89.062815 25019841

[b48] White D. S. Social Media Growth 2006 to 2012 (2013). Date of access: 2015.01.29.

[b49] Morrissey R. C., Goldman N. D. & Kennedy K. P. Skype S.A. United States Security Registration Statement, Amendment 3, Reg. No. 333-168646 (2011). Date of access: 2014.10.14.

[b50] Ruan Z., Iñiguez G., Karsai M. & Kertész J. Kinetics of social contagion. Phys. Rev. Lett. 115, 218702 (2015). 26636878 10.1103/PhysRevLett.115.218702

[b51] Dodds P. S. & Watts D. J. Universal Behavior in a Generalized Model of Contagion. Phys. Rev. Lett. 92, 218701 (2004). 15245323 10.1103/PhysRevLett.92.218701

[b52] Gleeson J. P. Binary-state dynamics on complex networks: Pair approximation and beyond. Phys. Rev. X 3, 021004 (2013).

[b53] Gleeson J. P. High-accuracy approximation of binary-state dynamics on networks. Phys. Rev. Lett. 107, 068701 (2011). 21902375 10.1103/PhysRevLett.107.068701

[b54] Brummitt C. D., Lee K.-M. & Goh K.-I. Multiplexity-facilitated cascades in networks. Phys. Rev. E 85, 045102(R) (2012). 10.1103/PhysRevE.85.045102 22680529

[b55] Lee K.-M., Brummitt C. D. & Goh K.-I. Threshold cascades with response heterogeneity in multiplex networks. Phys. Rev. E 90, 062816 (2014). 10.1103/PhysRevE.90.062816 25615156

[b56] Kobayashi T. Trend-driven information cascades on random networks. Phys. Rev. E 92, 062823 (2015). 10.1103/PhysRevE.92.062823 26764760

[b57] Bakshy E., Hofman J. M., Mason W. A. & Watts D. J. Everyone’s an influencer: Quantifying influence on Twitter. (WSDM ‘11, ACM, New York, NY, USA), pp. 65–74 (2011).

[b58] Porter M. A. & Gleeson J. P. Dynamical systems on networks: A tutorial. Eprint arXiv 1403.7663 (2014).

