# Supplementary material for: Local cascades induced global contagion: How heterogeneous thresholds, exogenous effects, and unconcerned behaviour govern online adoption spreading
Source: Sci Rep. 2016 Jun 7;6:27178. doi: 10.1038/srep27178 (PMC4895140; doi:10.1038/srep27178)
Supplement: Supplementary Information [file srep27178-s1.pdf]

Supplementary Information for

Local cascades induced global contagion: How heterogeneous thresholds, exogenous effects, and unconcerned behaviour govern online adoption spreading

M. Karsai*, G. Iñiguez, R. Kikas, K. Kaski, J. Kertész

*Corresponding author email: marton.karsai@ens-lyon.fr

Contents

S1 Detailed data description **2**

S2 Empirical determination of model parameters **2**

S2.1 Rate of innovators 2

S2.2 Degree distribution 3

S2.3 Threshold distribution 3

S3 Social influence - null model study **4**

S4 Threshold model **5**

S4.1 Model description 5

S4.2 Stochastic binary-state dynamics 6

S4.3 Reduced-dimension AMEs 7

S5 Waiting time of adoption **10**

S6 Empirical and model cluster statistics **11**

S7 Calculations for additional service **11**

S7.1 Empirical observations 11

S7.2 Model and validation 12

S1 Detailed data description

This study has been conducted on a dataset of the social network of Skype. The centrepiece of the dataset is the *contact network*, where nodes represent users and edges between pairs of users exist if they are in each other's contact lists. A user's contact list is composed of *friends*. If user u wants to add another user v to his/her contact list, u sends v a contact request, and the edge is established at the moment v approves the request (or not, if the contact request is rejected). Each edge is labelled with a time stamp indicating the moment the contact request was approved. As the underpinning social structure we consider the static representation of the Skype social network, aggregated for 99 months between September 2003 and November 2011. The largest connected component of this structure includes roughly 510 million users and 4.4 billion edges.

As the chosen service evolving on the Skype network, we follow how users purchase "credits" for calling phones. For each user, the dataset includes the date when he/she first adopted the paid product "buy credit" (first credit purchase, for all purposes). We select this service since its lifetime of 89 months is considerably long (it was introduced in 2004), and it can be adopted by registered Skype users only. This way the aggregated Skype network provides a complete description of the mediating social structure, which allows us to calculate the correct degree and adoption threshold for all individuals. To make additional observations and to further test our model, we perform calculations on a second paid service called "subscription", which was introduced in April 2008, lasts for over 42 months, and can also be adopted by registered Skype users only. Results regarding this service are presented in Section S7.

By considering the online social structure and the adoption times we identify users as innovator, vulnerable, or stable nodes based on the number Φ_k of adopting neighbours at the time of exposure. Thresholds are calculated as $\phi = \Phi_k/k$ for users with k contacts. The adoption network is constructed by considering confirmed social links between users who adopted the service earlier than the time of observation t. In order to avoid the effect of instantaneous group adoptions (evidently not driven by social influence), we only consider links between nodes who are neighbours in the underlying social network and whose adoption did not happen at the same time. Note that for the categorization of nodes we use only the adoption time and the state of their peers, and thus 'real' categories may differ slightly. For example, an innovator may appear as a vulnerable or stable node, even if its decision was not driven by social influence but some of its peers adopted earlier. To consider this bias we also measure 'effective' rates of adoption for the model process, just like for the empirical case (Fig.1, main text) and section S3.

The dataset does not include identity information. All usernames are anonymized and there is no way of inferring a user's identity solely from the profile. The dataset does not contain any information about interpersonal interactions, apart from the contact list.

S2 Empirical determination of model parameters

Parameters in the model are the rate of innovators p_n, the degree distribution $P(k)$, the threshold distribution $P(\phi)$, and the fraction of immune nodes r. Other than r, all of them can be estimated from the data as follows.

S2.1 Rate of innovators

As discussed in the main text, the rate of spontaneous adoption saturates approximately to a constant value after an initial transition period, which allows us to determine the rate of innovators by fitting a constant function on the curve after time 2τ. We estimate this rate to be $p_n = 0.00019$, as demonstrated in Fig. S2a where the dashed line assigns the fitted constant function.

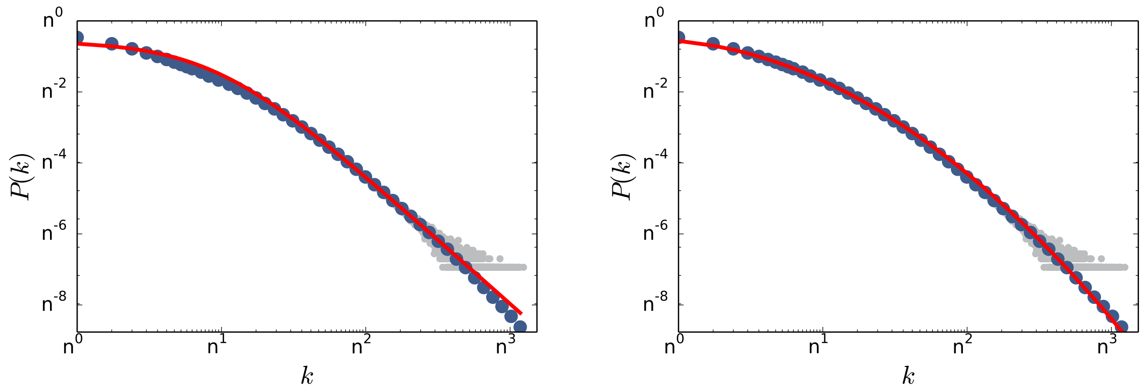

(a) **(b)**

Figure S1. Fitted degree distribution. **(a)** Empirical degree distribution $P(k)$ fitted with a shifted power-law distribution function [Eq. (S1)] with parameters described in the text. **(b)** $P(k)$ fitted by a lognormal distribution function [Eq. (S2)] with parameters determined in the text. Grey symbols are the degree distribution, blue symbols the log-binned distribution, and solid lines the fitted analytical distribution.

S2.2 Degree distribution

Degrees in the aggregated Skype network are broadly distributed with a fat tail corresponding to strong degree heterogeneities. To characterize this distribution analytically we select two candidate distribution functions. The first is a shifted power-law distribution function of the form,

$$P(k) = \frac{\gamma - 1}{C + k_{min}} \left(\frac{C + k}{C + k_{min}} \right)^{-\gamma} \quad \text{for} \quad k_{min} \leq k, \tag{S1}$$

where k denotes the degree, γ is the power-law exponent scaling the tail of the distribution, and k_{min} is the minimum degree (in our case 1). C is a constant scaling the shift of the distribution, which can be determined as $C = z(\gamma - 2) - k_{min}(\gamma - 1)$ since we know the average degree $z = 8.56$ of the empirical network. This way our only free parameter during the fit is the degree exponent γ. After fitting this function by using the non-linear least-square method, we obtain a relatively good match with the empirical distribution (Fig. S1a) for exponent $\gamma = 3.61$.

Our second candidate function is a lognormal distribution function of the form,

$$P(k) = \frac{1}{k\sigma_D \sqrt{2\pi}} e^{-\frac{(\ln k - \mu_D)^2}{2\sigma_D^2}} \quad \text{for} \quad k_{min} \leq k, \tag{S2}$$

where μ_D and σ_D are the scaling parameters. After fitting this function by using the non-linear least square method with two free parameters (μ_D and σ_D), we obtain an excellent fit with the empirical distribution for parameters $\mu_D = 1.2$ and $\sigma_D = 1.39$.

To select the best candidate function, we calculate the corresponding Jensen-Shannon (JS) divergence values [2] between the empirical and fitted distributions. As a result we find that for the shifted power-law function the best fit provides $JS = 0.0257$, while for the lognormal distribution we get $JS = 0.0051$. Thus we select the lognormal distribution as the best analytical function describing the degree distribution of the empirical network.

S2.3 Threshold distribution

The adoption threshold ϕ of a node is defined as $\phi = \Phi_k/k$, i.e. the fraction of adopting neighbours that trigger the adoption of the central node. Therefore it can only take certain fractional values determined by the degree k. Although thresholds are defined as a fraction, by considering nodes of the same degree

we can focus on the integer threshold Φ_k, defined as the number of a node's neighbours who have adopted the service earlier.

In our method we first group nodes of the same degree, record their integer thresholds, and then calculate the threshold distribution for each degree group, as shown in the main text (Fig. 1e, inset). These distributions collapse to a master curve after normalization by using the scaling relation $P(\Phi_k, k) = kP(\Phi_k/k)$ (Fig. 1e, main panel). Moreover, this master curve can be well approximated by a lognormal distribution of the form,

$$P(\phi) = \frac{1}{\phi\sigma_T\sqrt{2\pi}} e^{-\frac{(\ln\phi - \mu_T)^2}{2\sigma_T^2}}, \tag{S3}$$

where $\mu_T = -2$ and $\sigma_T = 1$, as determined by the empirical average threshold $w = 0.19$ and standard deviation (STD) 0.233.

These findings indicate that although individual thresholds are strongly determined by degree, their distribution is degree-invariant, suggesting that the fraction of adopting friends rather than its absolute number is relevant during the service adoption process. The estimated empirical values of parameters are summarized in Table 1.

p_n	$\langle k\rangle$	μ_D	σ_D	w	$STD(\phi)$	μ_T	σ_T
0.00019	8.56	1.2	1.39	0.19	0.233	-2	1

Table S1. Estimated empirical parameters for service "buy credit".

S3 Social influence - null model study

Studies of social contagion phenomena assume that social influence is responsible for the correlated adoption of connected people. However, an alternative explanation for the observed correlated adoption patterns is homophily: a link creation mechanism by which similar egos get connected in a social structure. In the latter case, the correlated adoption of a connected group of people would be explained by their similarity and not necessarily due to social influence. Homophily and influence are two processes that may simultaneously play a role during the adoption process. Nevertheless, distinguishing between them on the individual level is very difficult using our or any similar datasets [3]. Fortunately, at the system level one may decide which process is dominant in the empirical data. To do that first we need to elaborate on the differences between these two processes.

Influence-driven adoption of an ego can happen once one or more of its neighbours have adopted, since then their actions may influence the decision of the central ego. Consequently, the time ordering of adoptions of the ego and its neighbours matters in this case. Homophily-driven adoption is, however, different. Homophily drives social tie formation such that similar people tend to be connected in the social structure. In this case connected people may adopt because they have similar interests, but the time ordering of their adoptions would not matter. Therefore, it is valid to assume that adoption could evolve in clusters due to homophily, but these adoptions would appear in a random order.

To test our hypothesis we define a null model where we take the adoption times of each adopter and shuffle them randomly among all adopting egos. This way a randomly selected time is assigned to each adopter, while the adoption rate and the final set of adopters remain the same. Moreover, this procedure only destroys correlations between adoption events induced by social influence, but keeps the social network structure and node degrees unchanged. In this way, during the null model process the same egos appear as adopters, but the time series of adoption may in principle change (or not), corresponding to social influence (or homophily) as a dominant factor during the adoption process. If adoption is mostly driven by homophily, the rates of adoption would not change considerably beyond statistical fluctuations. On the other hand, if social influence plays a role in the process, rates of adoption in the null model should be very different from the empirical curves, implying that the time ordering of events matters in the adoption process. In this case, the rate of innovators should be higher than in the

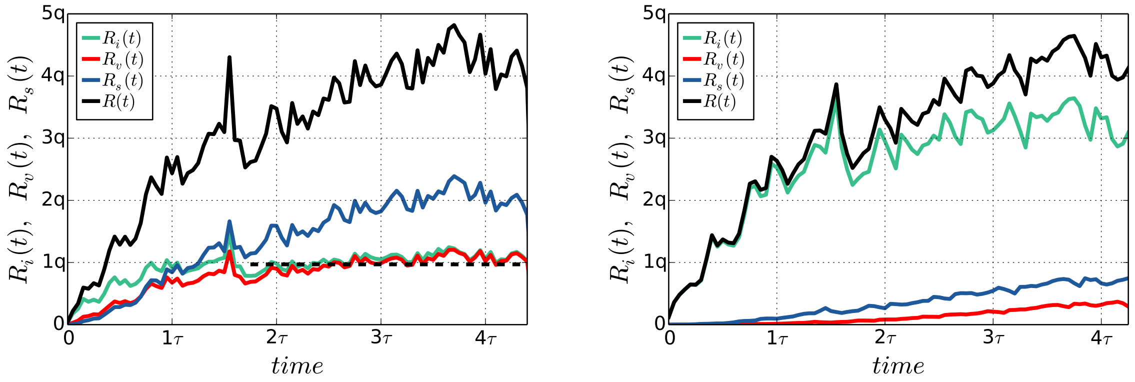

(a) Empirical adoption rates

(b) Null model adoption rates

Figure S2. Adoption rates in the original and null model processes. Adoption rates for innovator (green), vulnerable (red), and stable (blue) nodes as a function of time. **(a)** Empirical rates where adoptions appear in the original order. The dashed line assigns a fitted constant function to estimate the innovator adoption rate. **(b)** Null model rates where times of adoption are randomly shuffled. Here q and τ are arbitrary constant values.

empirical data, since nodes that are in the adoption cluster originally but not directly connected would have a greater chance to appear as innovators, due to a random adoption time that is not conditional to the time ordering of the adopting neighbours.

After calculating the adoption rates of different user groups in the shuffled null model sequence we observe the latter situation: the rate of innovators becomes dominant, while the rate of stable and vulnerable adoptions drops considerably as they appear only by chance. This suggests that the temporal ordering of adoption events matters a lot in the evolution of the observed adoption patterns, and thus social influence may play a strong role here. Of course one cannot decide whether influence is solely driving the process or homophily has some impact on it; in reality it probably does to some extent. However, we can use this null model measure to demonstrate the presence and importance of the mechanism of influence during the adoption process.

S4 Threshold model

S4.1 Model description

This model emulates the rise and temporal evolution of system-wide adoption cascades in complex social networks [4, 5, 6]. Note that this model has been introduced in [6], where its general scaling behaviour has been explored. In a system of fixed size, a node has social interactions with k other agents and is characterized by a continuous adoption threshold ϕ. When faced with the prospect of adopting a given innovation, product, or fad, susceptible individuals adopt spontaneously with rate p_n. Otherwise, the node adopts if at least a fraction ϕ of its k neighbours have adopted before (the so-called 'threshold rule'). Moreover, a fraction r of the system is 'immune' to the innovation, in the sense that these agents never adopt regardless of their values of k and ϕ. The distributions of degrees and thresholds, $P(k)$ and $P(\phi)$ (as well as the values of p_n and r), thus determine the average topological state and dynamical evolution of the system.

The model may be implemented numerically via a Monte Carlo simulation of the rules described above in a system of size N. Here, the dynamical state of the system is determined by the adoption state (0 or 1) of all nodes, which change in asynchronous random order in a series of time steps. Once an agent adopts and its state changes from 0 to 1, it remains so for the rest of the dynamics, thus ensuring a frozen final state for the finite system where no more adoptions arise. Each time step consists of N node updates: In each node update, a randomly selected node (non-immune and in state 0) adopts spontaneously with probability $p_r = p_n/(1-r)$ [1]; else it adopts only if the threshold rule is satisfied. The

[1] We define $p_r = 1$ for $p_n > 1 - r$.

rescaled rate p_r is necessary if we wish to obtain a rate p_n of innovators in early times of the dynamics, regardless of the value of r. Finally, we assume that agents with $k = 0$ receive no social influence (for any value of ϕ), and therefore can only adopt spontaneously. We will now explore this dynamics with numerical simulations and a rate equation formalism.

S4.2 Stochastic binary-state dynamics

Here we extend an approximate master equation (AME) formalism for stochastic binary-state dynamics as developed recently by Gleeson [7, 8, 9, 10]. In a stochastic binary-state dynamics, each node in the network can take one of two possible states (susceptible or adopter in the language of innovation adoption) at any point in time, and state-switching happens randomly with probabilities that only depend on the current state of the updating agent and on the states of its neighbours. This general definition includes the threshold model described above as a special case. Such formalism considers configuration-model networks, that is, an ensemble of networks specified by the degree distribution $P(k)$ but otherwise maximally random [11].

All relevant properties used to describe a node are included in the vector $\mathbf{k} = (k, c)$, where $k = k_0, k_1, \dots k_{M-1}$ is the degree of the node and $c = 0, 1, \dots, M$ a dummy variable that labels its 'type', i.e. any other property that characterizes the node apart from its degree. In the case of our threshold model, $c = 0$ is the type of the fraction r of immune nodes, while $c \neq 0$ labels the type of all non-immune nodes with given threshold ϕ_c. The various values of $c \neq 0$ correspond then to different adoption thresholds ϕ_c. The integer M is the maximum number of degrees/types considered in the AME framework, which can be increased to improve the accuracy of the analytical approximation at the expense of speed in its numerical computation[2]. Any pair of nodes with identical values of $\mathbf{k}$ are considered equivalent in this level of description, forming a node class with the same average dynamics. Moreover, $P(k)$ and $P(\phi)$ can be generalized to the joint distribution $P(\mathbf{k})$ giving the probability that a randomly selected node has property vector $\mathbf{k}$ (i.e. degree k and type c). Here it is useful to define $P(c)$ as the distribution of all non-zero types, $c = 1, \dots, M$. If degrees and thresholds are chosen independently, like in our model, then $P(\mathbf{k}) = rP(k)$ for $c = 0$ and $P(\mathbf{k}) = (1 - r)P(k)P(c)$ for $c > 0$. The distribution $P(c)$ is, in other words, a discrete, rescaled version of the continuous threshold distribution $P(\phi)$.

In the language of innovation adoption, the dynamics of a node is determined by the number $m = 0, 1, \dots k$ of its neighbours that have already adopted when the node is deciding whether to adopt or not. During a small time interval dt, a susceptible node (in state 0) adopts with probability $F_{\mathbf{k},m}dt$, while an adopter (in state 1) becomes susceptible with probability $R_{\mathbf{k},m}dt$. The functions $F_{\mathbf{k},m}$ and $R_{\mathbf{k},m}$, known as infection and recovery rates, respectively, determine the temporal evolution of the node class $\mathbf{k}$. In the particular case of threshold models, a so-called monotone dynamics, $R_{\mathbf{k},m} = 0 \;\forall\, \mathbf{k}, m$ (since no adopters become susceptible again). As for $F_{\mathbf{k},m}$, the rules of spontaneous and threshold adoption imply,

$$F_{\mathbf{k},m} = \begin{cases} p_r & \text{if} \quad m < k\phi_c \\ 1 & \text{if} \quad m \geq k\phi_c \end{cases}, \quad \forall m \text{ and } k, c \neq 0, \tag{S4}$$

that is, a node adopts the innovation either spontaneously with rate p_r, or with probability 1 if its number of adopting neighbours equals or exceeds the integer threshold $\Phi_k = \lceil k\phi_c \rceil$. Immune nodes ($c = 0$) have an infection rate of $F_{(k,0),m} = 0 \;\forall k, m$, while for isolated nodes ($k = 0$) $F_{(0,c),0} = p_r \;\forall c \neq 0$. In other words, immune nodes never adopt, and isolated nodes can only adopt spontaneously. We note that $F_{\mathbf{k},m}$ is written in terms of $p_r = p_n/(1 - r)$, not p_n, in order to counter the trivial decrease in the rate of spontaneous adoption for non-zero r.

Let us now turn to the rate equations for our threshold model, called AMEs in the formalism by Gleeson. We denote by $s_{\mathbf{k},m}(t)$ the fraction of $\mathbf{k}$-class nodes that are susceptible at time t and have

[2] Explicitly, rather than using $k_0 = k_{\min}$, $k_{M-1} = N - 1$ and $M = N - k_{\min}$ (i.e, considering all possible degrees in the empirical/simulated network), we take a small $k_0 > k_{\min}$ and large $k_{M-1} < N - 1$, $M < N - k_{\min}$, with the other $M - 2$ degree values equidistantly distributed between k_0 and k_{M-1}, thus disregarding some degrees and gaining speed in the computation of the AMEs. Similarly, the M threshold values corresponding to nonzero types are uniformly distributed in the open interval $(0, 1)$.

m adopting neighbours. Therefore, the fraction of agents with property vector $\mathbf{k}$ that are adopters at time t is $\rho_{\mathbf{k}}(t) = 1 - \sum_{m=0}^{k} s_{\mathbf{k},m}(t)$, and the fraction of adopters in the system is $\rho(t) = \sum_{\mathbf{k}} P(\mathbf{k})\rho_{\mathbf{k}}(t)$. Here, the sum over classes means a sum over all degrees and types, i.e. $\sum_{\mathbf{k}} \bullet = \sum_k \sum_c \bullet$. Assuming a monotone dynamics ($R_{\mathbf{k},m} = 0$), the AMEs for $s_{\mathbf{k},m}$ can be written as [7, 8, 10],

$$\frac{ds_{\mathbf{k},m}}{dt} = -F_{\mathbf{k},m}s_{\mathbf{k},m} - \beta_s(k-m)s_{\mathbf{k},m} + \beta_s(k-m+1)s_{\mathbf{k},m-1}, \tag{S5}$$

where $m = 0, \ldots, k$, $s_{\mathbf{k},-1} \equiv 0$, $F_{\mathbf{k},m}$ follows Eq. (S4), and $\beta_s(t)$ (the rate at which edges between pairs of susceptible nodes transform to edges between a susceptible agent and an adopter) is given by,

$$\beta_s(t) = \frac{\sum_{\mathbf{k}} P(\mathbf{k}) \sum_m (k-m) F_{\mathbf{k},m} s_{\mathbf{k},m}(t)}{\sum_{\mathbf{k}} P(\mathbf{k}) \sum_m (k-m) s_{\mathbf{k},m}(t)}. \tag{S6}$$

If at time $t = 0$ we randomly choose a fraction $\rho(0) = \sum_{\mathbf{k}} P(\mathbf{k})\rho_{\mathbf{k}}(0)$ of nodes as seed for the adoption process, the initial conditions for Eq. (S5) are $s_{\mathbf{k},m}(0) = [1 - \rho_{\mathbf{k}}(0)]B_{k,m}[\rho(0)]$, with $\rho_{\mathbf{k}}(0)$ the initial fraction of adopters in class $\mathbf{k}$ and $B_{k,m}$ a binomial factor,

$$B_{k,m}(\rho) = \binom{k}{m} \rho^m (1-\rho)^{k-m}. \tag{S7}$$

The solution $s_{\mathbf{k},m}(t)$ of the AME system in Eq. (S5) provides a very accurate description of the dynamics of our model, yet its dimension (i.e. number of equations to solve) grows quadratically with the number of degrees and linearly with the number of threshold values considered. Fortunately, the AMEs for our model can be mapped to a reduced-dimension system with a derivation similar to the one used by Gleeson in the case of the Watts threshold model [4, 5].

S4.3 Reduced-dimension AMEs

To reduce the dimension of Eq. (S5), we need to consider system-wide quantities that are more aggregated than $s_{\mathbf{k},m}$. One of them is the probability that a randomly chosen node is an adopter, $\rho(t) = 1 - \sum_{\mathbf{k}} P(\mathbf{k}) \sum_m s_{\mathbf{k},m}(t)$, i.e. the fraction of adopters in the network. The other one is the probability that a randomly chosen neighbour of a susceptible node is an adopter, $\nu(t) = \sum_{\mathbf{k}} P(\mathbf{k}) \sum_m m s_{\mathbf{k},m}(t) / \sum_m k s_{\mathbf{k},m}(t)$.

We start by proposing an exact solution for the AME system in terms of the following ansatz,

$$s_{\mathbf{k},m}(t) = [1 - \rho_{\mathbf{k}}(0)]B_{k,m}[\nu(t)]e^{-p_r t} \quad \text{for } m < k\phi_c \text{ and } c \neq 0, \tag{S8}$$

and $s_{(k,0),m} = B_{k,m}(\nu)$ for $c = 0$, where $B_{k,m}$ follows Eq. (S7). The meaning of the ansatz in Eq. (S8) is quite intuitive and considers two processes. First, a susceptible agent with degree k and m adopting neighbours is not selected as part of the initial adoption seed with probability $1 - \rho_{\mathbf{k}}(0)$ and is connected to m adopters with the binomially distributed probability $B_{k,m}(\nu)$. Second, for $m < k\phi_c$ a susceptible node does not fulfill the threshold rule and can only adopt spontaneously with probability $e^{-p_r t}$, since the system is progressively been filled by adopters. Considering these two processes as independent we end up with the product in Eq. (S8). Finally, since immune nodes do not adopt and are distributed randomly over the network, $s_{(k,0),m}$ is determined only by a binomial factor.

The next step is to insert the ansatz (S8) into the AME system (S5) and derive a set of differential equations for the aggregated quantities ρ and ν. Taking the time derivative $\dot{s}_{\mathbf{k},m}$ of Eq. (S8) (i.e. the left-hand side of Eq. (S5)) we get,

$$\dot{s}_{\mathbf{k},m} = \left(\left[\frac{m}{\nu} - \frac{k-m}{1-\nu} \right] \dot{\nu} - p_r \right) s_{\mathbf{k},m}. \tag{S9}$$

Then, we use the threshold rule (S4) for $m < k\phi_c$, the ansatz (S8) and the binomial identity,

$$B_{k,m-1}(\nu) = \frac{1-\nu}{\nu} \frac{m}{k-m+1} B_{k,m}(\nu), \tag{S10}$$

in the right-hand side of Eq. (S5) to obtain,

$$-F_{\mathbf{k},m}s_{\mathbf{k},m} - \beta_s(k-m)s_{\mathbf{k},m} + \beta_s(k-m+1)s_{\mathbf{k},m-1} = \left[-p_r + \beta_s\left(m - k + \frac{1-\nu}{\nu}m\right)\right]s_{\mathbf{k},m}. \quad \text{(S11)}$$

Equating Eqs. (S9) and (S11) as in the AME system (S5) leads to,

$$\dot{\nu} = \beta_s(1-\nu), \quad \text{(S12)}$$

a condition on ν so that the ansatz (S8) is a solution of Eq. (S5). This differential equation has the initial condition $\nu(0) = \rho(0)$, obtained by evaluating Eq. (S8) at $t = 0$ and comparing with the expression $[1 - \rho_{\mathbf{k}}(0)]B_{k,m}[\rho(0)]$, which corresponds to a random distribution of initial adopters among $\mathbf{k}$ classes. Furthermore, by assuming a (yet to be determined) function $g(\nu, t)$ such that $\dot{\nu} = g(\nu, t) - \nu$, Eq. (S12) reduces to,

$$\beta_s = \frac{g(\nu,t) - \nu}{1-\nu}. \quad \text{(S13)}$$

Now, we consider the following general result derived by Gleeson in [8] (Eqs. (F6)–(F10) therein),

$$\sum_{\mathbf{k}} P(\mathbf{k}) \sum_m (k-m)s_{\mathbf{k},m} = z(1-\nu)^2, \quad \text{(S14)}$$

with $z = \sum_k kP(k)$ the average degree in the network. Eq. (S14) is valid for functions $s_{\mathbf{k},m}$ and ν that satisfy Eqs. (S5) and (S12) respectively, for any $F_{\mathbf{k},m}$ and random initial conditions on $s_{\mathbf{k},m}$ and ν, and is thus applicable in our case. Our goal here is to use Eq. (S14) to find an expression for $g(\nu)$ and therefore write the differential equation (S12) explicitly. Noting that the left-hand side of Eq. (S14) is the denominator in the definition (S6) of β_s and that $F_{(k,0),m} = 0$ (i.e. immune nodes do not adopt), Eq. (S6) gives,

$$\begin{aligned}\beta_s &= \frac{1-r}{z(1-\nu)^2}\left[p_r \sum_{\mathbf{k}|c\neq 0} P(k)P(c) \sum_{m<k\phi_c} (k-m)s_{\mathbf{k},m} + \sum_{\mathbf{k}|c\neq 0} P(k)P(c) \sum_{m\geq k\phi_c} (k-m)s_{\mathbf{k},m}\right] \\ &= \frac{1}{z(1-\nu)^2}\left[\sum_{\mathbf{k}} P(\mathbf{k}) \sum_m (k-m)s_{\mathbf{k},m} - r\sum_k P(k)\sum_m (k-m)s_{(k,0),m} \right. \\ &\quad \left. -(1-r)(1-p_r)\sum_{\mathbf{k}|c\neq 0} P(k)P(c) \sum_{m<k\phi_c} (k-m)s_{\mathbf{k},m}\right], \end{aligned} \quad \text{(S15)}$$

where we have written $P(\mathbf{k})$ explicitly as $P(\mathbf{k}) = rP(k)$ for $c = 0$ and $P(\mathbf{k}) = (1-r)P(k)P(c)$ for $c > 0$, in order to notice the dependence on r. Then, we insert the ansatz (S8) (with its special case $s_{(k,0),m} = B_{k,m}(\nu)$ for immune nodes), as well as the identities $(k-m)B_{k,m}(\nu) = k(1-\nu)B_{k-1,m}(\nu)$ and $\sum_{m<k\phi_c} B_{k-1,m}(\nu) = 1 - \sum_{m\geq k\phi_c} B_{k-1,m}(\nu)$ to obtain,

$$\begin{aligned}\beta_s = \frac{1}{1-\nu}\Bigg((1-r)\Bigg[&1 - (1-p_r)e^{-p_r t} \\ &+(1-p_r)e^{-p_r t}\sum_{\mathbf{k}|c\neq 0}\frac{k}{z}P(k)P(c)\left(\rho_{\mathbf{k}}(0) + [1-\rho_{\mathbf{k}}(0)]\sum_{m\geq k\phi_c} B_{k-1,m}(\nu)\right)\Bigg] - \nu\Bigg). \end{aligned} \quad \text{(S16)}$$

A comparison of Eqs. (S13) and (S16) gives us the following expression for $g(\nu, t)$,

$$g(\nu,t) = (1-r)\left(f_t + (1-f_t)\sum_{\mathbf{k}|c\neq 0}\frac{k}{z}P(k)P(c)\left[\rho_{\mathbf{k}}(0) + [1-\rho_{\mathbf{k}}(0)]\sum_{m\geq k\phi_c} B_{k-1,m}(\nu)\right]\right), \quad \text{(S17)}$$

where we define f_t as $f_t = 1 - (1-p_r)e^{-p_r t}$. Thus, the AME system (S5) gets reduced to the differential equation $\dot{\nu} = g(\nu, t) - \nu$, with $g(\nu, t)$ given explicitly by Eq. (S17).

Even though the equation $\dot{\nu} = g(\nu, t) - \nu$ is closed and in this sense equivalent to Eq. (S5), we can also derive the corresponding equation for ρ, since we are mainly interested in the temporal evolution of the fraction of adopters in the network. From the definition of ρ and Eq. (S5) we have,

$$\begin{aligned}\dot{\rho} = -\sum_{\mathbf{k}} P(\mathbf{k}) \sum_m \dot{s}_{\mathbf{k},m} = \sum_{\mathbf{k}} P(\mathbf{k}) \sum_m F_{\mathbf{k},m} s_{\mathbf{k},m} \\ + \beta_s \sum_{\mathbf{k}} P(\mathbf{k}) \sum_m \big[(k-m)s_{\mathbf{k},m} - (k-m+1)s_{\mathbf{k},m-1}\big],\end{aligned} \tag{S18}$$

where the second term in the right-hand side telescopes to zero. Then, we use an algebraic manipulation similar to that of Eqs. (S15) and (S16) to obtain,

$$\begin{aligned}\sum_{\mathbf{k}} P(\mathbf{k}) \sum_m F_{\mathbf{k},m} s_{\mathbf{k},m} &= (1-r)\left(p_r \sum_{\mathbf{k}|c\neq 0} P(k)P(c) \sum_{m<k\phi_c} s_{\mathbf{k},m} + \sum_{\mathbf{k}|c\neq 0} P(k)P(c) \sum_{m\geq k\phi_c} s_{\mathbf{k},m} \right) \\ &= (1-r)\left(1 - (1-r)(1-p_r) \sum_{\mathbf{k}|c\neq 0} P(k)P(c) \sum_{m<k\phi_c} s_{\mathbf{k},m} \right) - \rho \\ &= (1-r)\left(f_t + (1-f_t) \sum_{\mathbf{k}|c\neq 0} P(k)P(c) \left[\rho_{\mathbf{k}}(0) + [1-\rho_{\mathbf{k}}(0)] \sum_{m\geq k\phi_c} B_{k,m}(\nu) \right] \right) - \rho.\end{aligned} \tag{S19}$$

In this way, Eqs. (S18) and (S19) can be rewritten as $\dot{\rho} = h(\nu, t) - \rho$, where,

$$h(\nu,t) = (1-r)\left(f_t + (1-f_t) \sum_{\mathbf{k}|c\neq 0} P(k)P(c) \left[\rho_{\mathbf{k}}(0) + [1-\rho_{\mathbf{k}}(0)] \sum_{m\geq k\phi_c} B_{k,m}(\nu) \right] \right). \tag{S20}$$

Joining all of these results, the AME system (S5) gets reduced to the system of two ordinary differential equations,

$$\dot{\rho} = h(\nu, t) - \rho, \tag{S21a}$$

$$\dot{\nu} = g(\nu, t) - \nu, \tag{S21b}$$

with the quantities $g(\nu)$ and $h(\nu)$ given explicitly by Eqs. (S17) and (S20).

The system (S21) can be solved numerically to obtain $\rho(t)$ and thus characterize the temporal evolution of the adoption process. Let us further separate the fraction of adopters as $\rho(t) = \rho_0(t) + \rho_1(t)$, where ρ_0 and ρ_1 are the fractions of innovators and induced adopters (i.e. vulnerable and stable nodes), respectively. Now consider the identity,

$$1 - \rho = \sum_{\mathbf{k}} P(\mathbf{k}) \sum_m s_{\mathbf{k},m} = r + (1-r) \sum_{\mathbf{k}|c\neq 0} P(k)P(c) \sum_m s_{\mathbf{k},m} = r + \rho_s, \tag{S22}$$

where $\rho_s(t)$ is the fraction of non-immune, susceptible nodes that can eventually adopt, either spontaneously or not. Since such susceptible nodes adopt spontaneously at a rate p_r, the rate equation for innovators is $\dot{\rho}_0 = p_r \rho_s$. Then, with Eq. (S22) we obtain,

$$\rho_0(t) = p_r \int_0^t [1 - r - \rho(t)] dt, \tag{S23}$$

which can be calculated explicitly with the numerical solution of Eq. (S21).

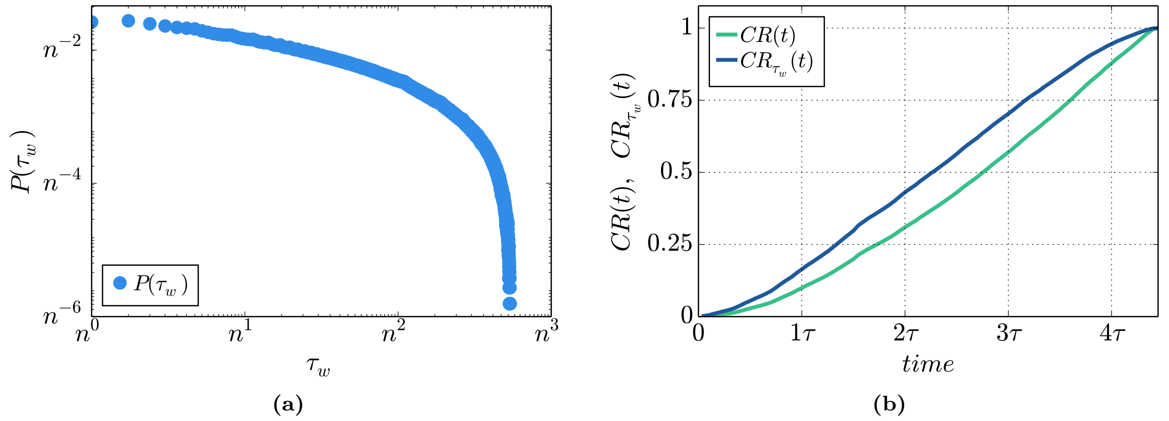

Figure S3. Waiting time distribution and its effect on the adoption process (a) Distribution $P(\tau_w)$ of times between the last adoption in the egocentric network of an individual and his/her own adoption. **(b)** Cumulative adoption rates after waiting time removal [$CR(t)$ and $CR_{\tau_w}(t)$, respectively]. n and τ are arbitrary constant values.

S5 Waiting time of adoption

Another reason behind the non-rapid evolution of the adoption process could be the time users wait after their personal adoption threshold is reached and before adopting the service. This lag in adoption can be due to individual characteristics, or come from the fact that social influence does not spread instantaneously (as commonly assumed in threshold models, including ours). However, the waiting time τ_w can be estimated by measuring the time difference between the last adoption in a user's egocentric network and the time of adoption. We define $\tau_w = 0$ for innovators, but τ_w can take any positive value for vulnerable and stable adopters up to the length of the observation period.

Waiting times are broadly distributed for adopters (Fig. S3a), meaning that many users adopt the service shortly after their personal threshold is reached, but a considerable fraction waits long before adopting the service. The heterogeneous nature of waiting times may be a key element behind the observed adoption dynamics. One way to figure out the effect of waiting times on the speed of cascade evolution is by removing them. We can extract waiting times from adoption times and thus calculate rescaled adoption times. The rescaled adoption time of a user is the last time when his/her fraction of adopting neighbours changed and the adoption threshold was (hypothetically) reached. After this procedure we can calculate a new adoption rate function by using rescaled adoption times and compare it to the original. From Fig. S3b we can conclude that although adoption becomes faster, the rescaled adoption dynamics is still not rapid. On the contrary, it suggests that the rescaled adoption dynamics is still very slow and quite similar to the original. Consequently, waiting times cannot explain the observed dynamics of adoption.

Note that long waiting times can have a further effect on the measured dynamics. After the 'real' threshold of a user is reached and he/she waits to adopt, some neighbours may adopt the product. Hence all observed measures are in this sense 'effective': observed thresholds are larger or equal than real thresholds; the innovator rate is smaller or equal; the vulnerable and stable rates will be larger or equal; and waiting times will be shorter or equal than the real values. Consequently the process may be actually faster than that we observe in Fig. S3b after removing effective waiting times. However, this bias becomes important only after the majority of individuals in the social network has adopted the service and the spontaneous emergence of adopting neighbours becomes more frequent. As the fraction of adopters in our dataset is always less than 6% [12], we expect minor effects of this observational bias on measurements.

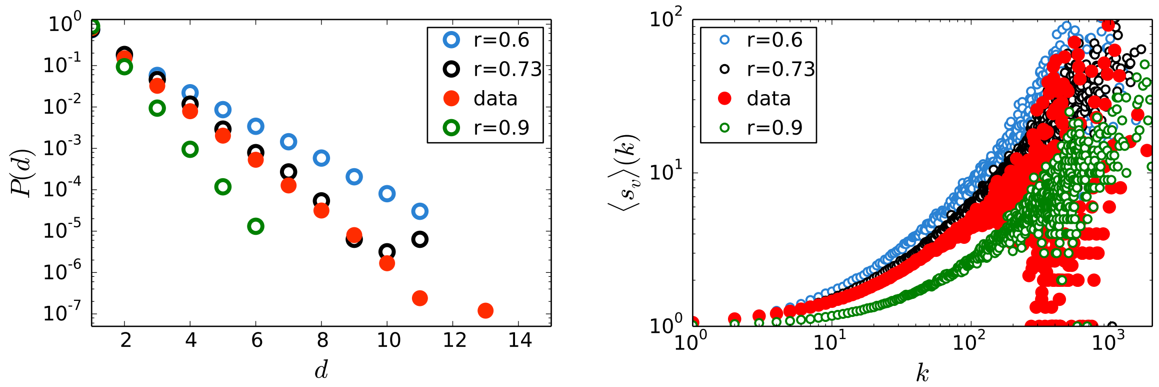

(a) **(b)**

Figure S4. Empirical and model cluster statistics (a) Distribution $P(d)$ of the depth of induced vulnerable trees in the empirical and model systems. **(b)** Correlation $\langle s_v \rangle(k)$ between the degree of innovators and the average size of vulnerable trees they induce. Empty symbols denote model calculations for $r = 0.6$ (blue), 0.73 (black), and 0.9 (green), and full red symbols the empirical measurements. Model calculations correspond to networks of size $N = 10^6$ and are averaged over 100 independent realizations.

S6 Empirical and model cluster statistics

As described in the main text, we perform extensive model calculations using empirically determined parameters to estimate the only unknown parameter, the fraction of immune nodes r. We match the relative size of the largest connected component of the real adoption network with its corresponding measure in the model at the end of the observation period, and estimate the fraction of immune nodes in the real system as $r = 0.73$. To support our estimation we also measure the distribution $P(d)$ of the depth of induced vulnerable trees and the correlation $\langle s_v \rangle(k)$ between the degree of innovator nodes and the average size of induced vulnerable trees in the model, and match them with the equivalent empirical measures. To provide further support for the estimated r value we show the dependence of these quantities of different r values.

We measure $P(d)$ and $\langle s_v \rangle(k)$ for $r = 0.6$ and 0.9, as well as for the predicted value $r = 0.73$ (Fig. S4). It is clear that both quantities scale with r. For smaller r more nodes are susceptible for adoption, allowing deeper and larger vulnerable trees, while for larger r no large induced cluster can emerge as the system is forced into a quenched state. Moreover, measures for the estimated r value fit the empirical data considerably well. This collapse is remarkable, since we neglect any higher-order structural and temporal correlations in the model (like assortative mixing, community structure, bursty adoption patterns, periodic activity fluctuations, etc.), which are present in the empirical system. Differences in the tails of the measures are due to finite-size effects since the modelled network is two orders of magnitude smaller than the empirical social structure. Note that although we can look for an r fraction that produces a better fit between model and data in terms of $P(d)$ and $\langle s_v \rangle(k)$, the collapse in Fig. S4 demonstrates the quality of an independent procedure of estimating r (i.e. by matching the relative size of components). Therefore, these results are intended for validation only and not as a method to estimate the correct value of r.

S7 Calculations for additional service

S7.1 Empirical observations

In order to support our empirical observations and modelling of the social spreading of Skype, we examine the adoption dynamics of an additional paid service called "subscription", introduced in April 2008 and

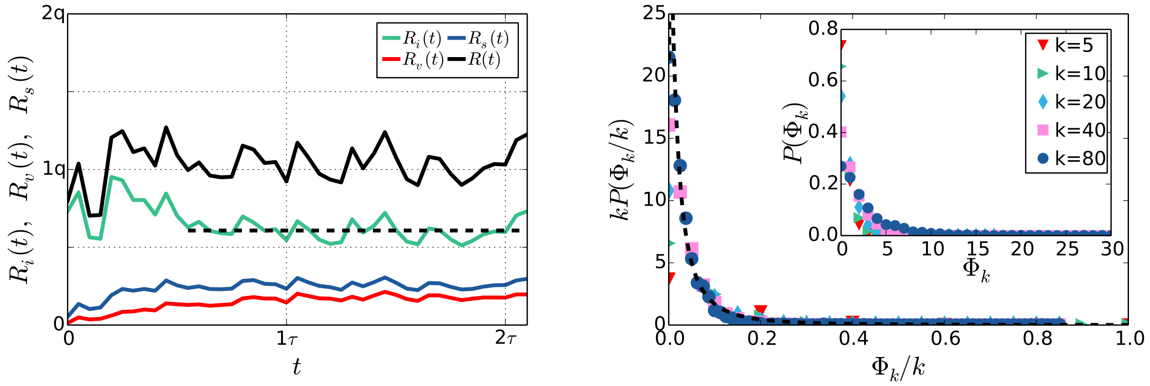

(a) **(b)**

Figure S5. Adoption rates and threshold distributions for service "subscription". **(a)** Net adoption rate (black), as well as rates for innovator (green), vulnerable (red), and stable (blue) nodes as function of time. The dashed line is a fitted constant function to estimate the innovator adoption rate as $p_n = 0.00012$. **(b)** Distribution of integer thresholds Φ_k for several degree groups (inset). By using $P(\Phi_k, k) = kP(\Phi_k/k)$ these curves collapse to a master curve well approximated by a lognormal function (dashed line) with average $w = 0.063$ and STD 0.153 (for further details see Section S2.3).

with adoption data for over 42 months until the end of the observation period. This service is only available for registered Skype users, and we can therefore use the accumulated static Skype network as background social structure. In order to investigate the adoption of this service we repeat all calculations described previously. First we measure the decoupled rate of innovator, vulnerable, and stable adopters (Fig. S5a). We see that after a short initial period innovators adopt approximately with a constant rate, setting the model parameter to $p_n = 0.00012$. Moreover, here innovators dominate social spreading since the rate of vulnerable and stable adoptions is relatively low.

We also measure the integer threshold distribution for different degree groups (Fig. S5b, inset) just as described in Section S2.3. These distributions scale together after normalization with the scaling relation $P(\Phi_k, k) = kP(\Phi_k/k)$ (Fig. S5b, main panel) and are well approximated by a lognormal distribution [Eq. (S3)] with parameters $\mu_T = -3.73$ and $\sigma_T = 1.39$, as determined by the average threshold $w = 0.063$ and STD 0.153. Note that since the adoption dynamics of this service is dominated by innovators, the average threshold w is smaller than in the case of the "buy credit" service. All parameters are summarized in Table 2. Since the background network is the same for both services, network parameters are those of Table.1.

Although the adoption process is dominated by innovators, a giant connected component evolves in the adoption network (Fig. S5a, main panel). On the other hand, its relative size is considerable smaller than for the "buy credit" service. The stable adoption network is also dominated by a giant component, but its relative size is even smaller when compared to the adoption network (Fig. S5a, inset). Moreover, the largest vulnerable trees are only two orders of magnitude smaller than the stable giant cluster (Fig. S5b). For comparison, this difference is five order of magnitude for the "buy credit" service.

p_n	w	$STD(\phi)$	μ_T	σ_T
0.00012	0.063	0.153	-3.73	1.39

Table S2. Estimated empirical parameters for service "subscription".

S7.2 Model and validation

We repeat all model calculations with the parameters of the "subscription" service to see whether we can recover its adoption dynamics by using the dynamical threshold model introduced in the main text and in

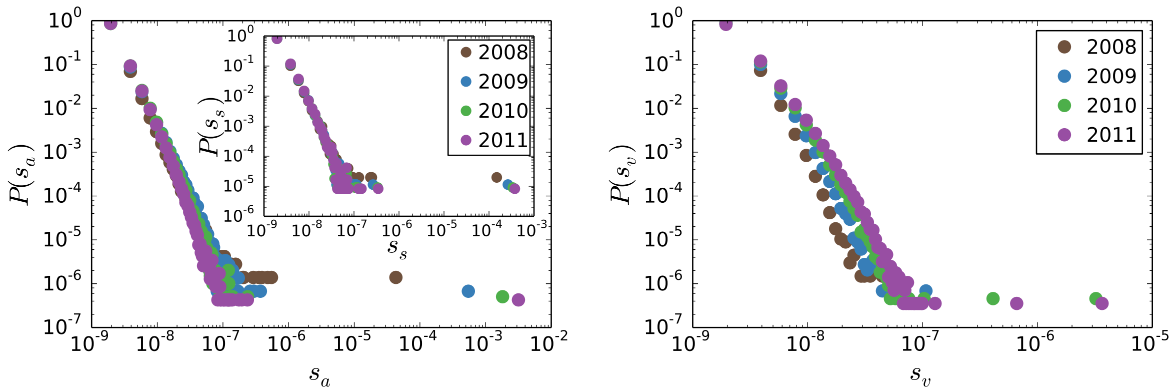

(a) **(b)**

Figure S6. Empirical cluster statistics. **(a)** Relative connected-component size distribution $P(s)$ at different times for the empirical adoption network (main panel) and the stable adoption network (inset), with sizes s_a and s_s, respectively. **(b)** Relative connected-component size distribution $P(s_v)$ of the empirical innovator-induced vulnerable trees at different times.

Section S4. We check the dependence on r of the average size of the largest connected component of the network (LC) of susceptible nodes available for the adoption process, the adoption network, the stable adoption network, and of vulnerable trees (Fig. S7, upper panel). In addition we record the average size LC^{2nd} of the second largest connected component (Fig. S7, middle panel). Finally we show the time when the adoption process has reached the 50% of available susceptible nodes in the adoption network (Fig. S7, lower panel).

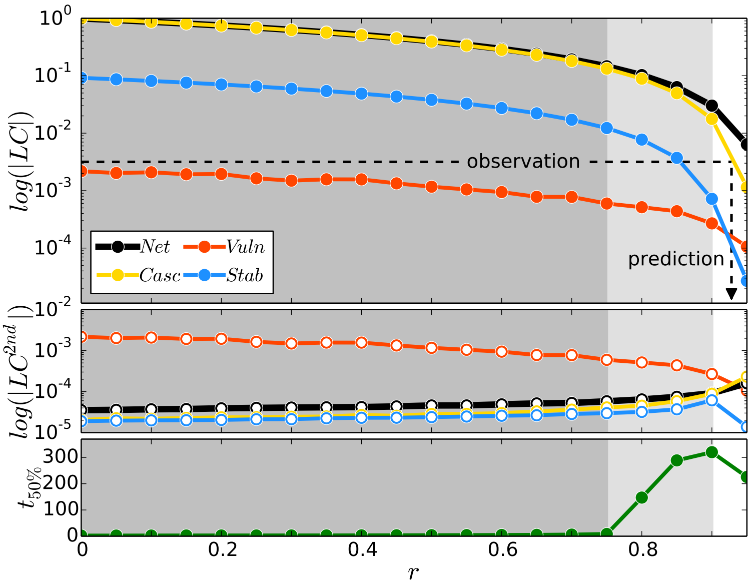

Figure S7. Modeled adoption process of the service "subscription" Average size of the largest (LC) and 2nd largest (LC^{2nd}) components of the model network ('Net'), model adoption network ('Casc'), model stable network ('Stab'), and induced vulnerable trees ('Vuln') as a function of r. Dashed lines show the observed relative size of the real LC of the adopter network in 2011 (Fig. S6, main panel) and the predicted r value. The lower panel depicts the time $t_{50\%}$ when the adoption process has reached 50% of the susceptible network as a function of r. We use 100 realizations of configuration-model networks with size $N = 10^5$ and lognormal degree distribution parametrized as described in Section S2.2. Model calculations correspond to the parameters of Table 2 for 42 iteration steps (matching the length of the observation period).

The r dependence of the adoption process appears to be qualitatively similar to our earlier calculations on the "buy credit" service, but there are remarkable differences. Firstly, the crossover regime (depicted by the light grey area in Fig. S7) is shifted towards larger r values due to the different threshold distribution and innovator adoption rate. Secondly, after matching the relative size of the largest connected component of the empirical adoption network (last point on the right-hand side of Fig. S6, main panel), the predicted $r = 0.928$ is out of the crossover regime. At this point the background social network is still not fragmented (as evidenced by the black line in Fig. S7, which has not reached its maximum yet) and it allows for the emergence of large connected adoption clusters. It is very sparse, however, which explains: (a) the dominating innovator adoption rate observed empirically; (b) the reduced size of the giant component of the adoption and stable adoption networks; and (c) the relatively large innovator trees as compared to the stable adoption network components. We observe that the largest vulnerable trees are smaller than the largest stable clusters in the empirical data, while the opposite is true for the model. A possible explanation of this difference is the assumption in the model that the network is degree-uncorrelated. This is a necessary approximation in order to treat the model analytically, but it might not hold for the empirical network. All in all, this picture suggests that the "subscription" service is out of the rapid and even the crossover cascading regimes, and that its dynamics is mostly driven by independent innovators rather than social influence, on a network of which a large majority is not susceptible to innovation.

References

[1] White D. S., *Social Media Growth 2006 to 2012* (2013). Date of access: 2015.01.29.

[2] Lin J., Divergence measures based on the Shannon entropy. *Trans. Inf. Theory* **37**, 145 (2009).

[3] Shalizi C. R. and Thomas A. C., Homophily and Contagion Are Generically Confounded in Observational Social Network Studies. *Sociol. Methods Res.* **40**(2), 211–239 (2011).

[4] Watts D. J., A simple model of global cascades on random networks. *Proc. Natl. Acad. Sci. USA* **99**, 5766–5771 (2002).

[5] Singh P., Sreenivasan S., Szymanski B. K., Korniss Gy., Threshold-limited spreading in social networks with multiple initiators. *Sci. Rep.* **3**, 2330 (2013).

[6] Ruan Z., Iñiguez G., Karsai M., Kertész J., Kinetics of social contagion. *Phys. Rev. Lett.* **115**, 218702 (2015).

[7] Porter M. A., Gleeson J. P., Dynamical systems on networks: A tutorial. Eprint arXiv 1403.7663 (2014).

[8] Gleeson J. P., Binary-state dynamics on complex networks: Pair approximation and beyond. *Phys. Rev. X* **3**, 021004 (2013).

[9] Gleeson J. P., Cascades on correlated and modular random networks. *Phys. Rev. E* **77**, 046117 (2008).

[10] Gleeson J. P., High-accuracy approximation of binary-state dynamics on networks. *Phys. Rev. Lett.* **107**, 068701 (2011).

[11] Newman M. E. J., *Networks: An Introduction.* (Oxford University Press) (2010).

[12] Morrissey R. C., Goldman N. D., Kennedy K. P,. Skype S.A. United States Security Registration Statement, Amendment 3, Reg.No. 333-168646 (2011). Date of access: 2014.10.14.
